# Nr2e1 regulates retinal lamination and the development of Müller glia, S-cones, and glycineric amacrine cells during retinogenesis

**DOI:** 10.1186/s13041-015-0126-x

**Published:** 2015-06-20

**Authors:** Ximena Corso-Díaz, Elizabeth M. Simpson

**Affiliations:** Centre for Molecular Medicine and Therapeutics at the Child and Family Research Institute, University of British Columbia, 950 W 28 Ave, Vancouver, V5Z 4H4 BC Canada; Genetics Graduate Program, University of British Columbia, Vancouver, V6T 1Z2 BC Canada; Department of Medical Genetics, University of British Columbia, Vancouver, V6T 1Z3 BC Canada; Department of Psychiatry, University of British Columbia, Vancouver, V6T 2A1 BC Canada

**Keywords:** Nr2e1, Amacrines, S-cones, Müller glia, Brn3a, Chimera, Ectopic plexiform layer

## Abstract

**Background:**

Nr2e1 is a nuclear receptor crucial for neural stem cell proliferation and maintenance. In the retina, lack of Nr2e1 results in premature neurogenesis, aberrant blood vessel formation and dystrophy. However, the specific role of Nr2e1 in the development of different retinal cell types and its cell-autonomous and non-cell autonomous function(s) during eye development are poorly understood.

**Results:**

Here, we studied the retinas of P7 and P21 *Nr2e1*^*frc/frc*^ mice and *Nr2e1*^*+/+*^ ↔ *Nr2e1*^*frc/frc*^ chimeras. We hypothesized that Nr2e1 differentially regulates the development of various retinal cell types, and thus the cellular composition of *Nr2e1*^*frc/frc*^ retinas does not simply reflect an overrepresentation of cells born early and underrepresentation of cells born later as a consequence of premature neurogenesis. In agreement with our hypothesis, lack of *Nr2e1* resulted in increased numbers of glycinergic amacrine cells with no apparent increase in other amacrine sub-types, normal numbers of Müller glia, the last cell-type to be generated, and increased numbers of *Nr2e1*^*frc/frc*^ S-cones in chimeras. Furthermore, *Nr2e1*^*frc/frc*^ Müller glia were mispositioned in the retina and misexpressed the ganglion cell-specific transcription factor Brn3a. *Nr2e1*^*frc/frc*^ retinas also displayed lamination defects including an ectopic neuropil forming an additional inner plexiform layer. In chimeric mice, retinal thickness was rescued by 34 % of wild-type cells and *Nr2e1*^*frc/frc*^ dystrophy-related phenotypes were no longer evident. However, the formation of an ectopic neuropil, misexpression of Brn3a in Müller glia, and abnormal cell numbers in the inner and outer nuclear layers at P7 were not rescued by wild-type cells.

**Conclusions:**

Together, these results show that Nr2e1, in addition to having a role in preventing premature cell cycle exit, participates in several other developmental processes during retinogenesis including neurite organization in the inner retina and development of glycinergic amacrine cells, S-cones, and Müller glia. Nr2e1 also regulates various aspects of Müller glia differentiation cell-autonomously. However, Nr2e1 does not have a cell-autonomous role in preventing retinal dystrophy. Thus, Nr2e1 regulates processes involved in neurite development and terminal retinal cell differentiation.

**Electronic supplementary material:**

The online version of this article (doi:10.1186/s13041-015-0126-x) contains supplementary material, which is available to authorized users.

## Background

During retinal development, six neuronal cell types and one type of glia are generated in a conserved histogenic yet overlapping order [1]. Ganglion neurons are the first cells to be generated followed by amacrine, horizontal, cones, and rods during the embryonic period, and bipolar and Müller glia during the postnatal period [1]. These cell types are organized into three nuclear layers and generate two plexiform layer neuropils where most of the synapses are confined. Retinal progenitor cell (RPC) proliferation and fate choice are regulated by intrinsic mechanisms [2–4] and extracellular signaling from differentiated cells [5–7].

During retinogenesis, the cell-cycle length increases over time in progenitor cells [8]. In various vertebrates, including mice, premature cell cycle exit in the retina increases the number of cells that are born early and decreases the number of cells born towards the end of retinal histogenesis [9–12]. RPCs are heterogeneous in gene expression [13] and express transcription factors and cell cycle regulators that play dual roles in controlling RPC cell cycle and fate [12, 14, 15].

Nr2e1 is a conserved orphan nuclear receptor that regulates neural stem cell proliferation and maintenance [16]. *Nr2e1*-null mice have smaller brains and retinas, are blind, and highly aggressive [17]. Nr2e1 is mostly a repressor that operates in a cell-autonomous fashion, but it can also activate gene transcription [18, 19] and act non-cell-autonomously through the Wnt pathway to regulate neural stem cell proliferation and self-renewal [19]. Lack of *Nr2e1* results in premature cell cycle exit during corticogenesis and reduced thickness of superficial cortical layers due to a depletion of the neural stem cell pool [16]. Lack of Nr2e1 in the retina results in precocious neurogenesis, impaired blood vessel development [20], and progressive dystrophy [21, 22]. This complex phenotype poses a challenge to understanding the role of Nr2e1 in specific retinal cell populations.

Chimeras provide valuable information regarding the autonomous and non-autonomous cellular consequences of gene mutations, the development of different cell-types and their interaction through cell-signaling, as well as the nature of tissue-tissue interactions in vivo [23]. To better understand the role(s) of Nr2e1 in retinal development, we studied the cellular composition and morphology of *Nr2e1*^*frc/frc*^, and *Nr2e1*^*+/+*^↔*Nr2e1*^*frc/frc*^ chimeric mouse retinas. We found that dystrophy-related phenotypes in *Nr2e1*^*frc/frc*^ retinas are not generated cell-autonomously. In addition, we found that lack of *Nr2e1* results in an ectopic plexiform layer in the inner retina, aberrant development of Müller glia and a bias towards the generation of glycinergic amacrine cells, S-cones and Müller glia.

## Results

To get insight into the cell autonomy of Nr2e1 during retinogenesis we used *Nr2e1*^*frc/frc*^ and chimeric mice comprised of both *Nr2e1*^*frc/frc*^ and wild-type cells. We studied abnormal phenotypes previously reported to be present in *Nr2e1* null retinas, such as reduced retinal thickness and blood vessel numbers. We later focused on the role of Nr2e1 in cell type development by studying the numbers and localization of different cell types.

### Expression of EGFP and β-galactosidase in mouse chimeras

To better understand the cell-autonomous and non-cell autonomous roles of Nr2e1 during retinogenesis, we made chimeric mice comprised of *Nr2e1*^*+/+*^ and *Nr2e1*^*frc/frc*^ cells, herein referred as Wt↔*frc* chimeras. Experimental and control chimeric mice were made by blastocyst injection of *Nr2e1*^*+/+*^ or *Nr2e1*^*frc/frc*^ embryonic stem cells (ESCs) harboring a ubiquitous-expressing EGFP transgene (Additional file 1: Figure S1A and B). In contrast, host blastocyst contained the *lacZ* gene under the control of *ROSA26* promoter (*R26*^*lacZ*^) thus expressing β-galactosidase (β-gal) (Additional file 1: Figure S1B). In this way ESC-derived cells could be identified by the green epifluorescence of EGFP and blastocyst-derived cells by the enzymatic product of β-gal.

We used two different embryonic stem cell lines per genotype to control for possible cell-line specific traits that could affect the phenotype of the mice. Four Wt↔Wt and four Wt↔*frc* chimeras were studied at P7. Nine Wt↔Wt and ten Wt↔*frc* chimeras were studied at P21. Eyes from these chimeras were subjected to funduscopy and collected for cryosectioning. First, we determined that the EGFP and β-gal markers were expressed appropriately in the chimeras. We assessed the expression of β-gal by its enzymatic activity and could clearly observe the blue precipitate formed by the hydrolysis of X-gal in perinuclear regions (Additional file 1: Figure S1C). Importantly, this enzymatic reaction did not interfere with the EGFP epifluorescence and both markers were expressed in mutually exclusive regions of the chimeric retinas (Additional file 1: Figure S1D). We assessed the percentage of chimerism by measuring the area displaying EGFP epifluorescence in the ONL plus INL of each retina and comparing it to the total ONL plus INL area. We excluded the IPL and GCL to decrease the interfering signal recovered from neural processes. Thus, we were able to use these two markers reliably as indicators of the origin of the different cell types; ESC or host blastocyst.

### *Nr2e1*^*frc/frc*^ reduced retinal thickness and blood vessel numbers were rescued in Wt↔*frc* chimeras

Next, we assessed whether the reduced retinal thickness and blood vessel numbers of *Nr2e1*^*frc/frc*^ retinas could be rescued by wild-type cells and how many wild-type cells would be needed to achieve rescue. Detailed information for each chimera is given in Table 1.

**Table 1:** Characteristics of the chimeras

ID	Age	Chimerism %	ESC line	Genotype	Blood vessel #	Retinal thickness (μM)
7586	P7	48	mEMS4922	*Nr2e1* ^*frc/frc*^	na	213.63 ± 9.36
7585	P7	58	mEMS4922	*Nr2e1* ^*frc/frc*^	na	234.90 ± 13.84
7577	P7	62	mEMS4914	*Nr2e1* ^*frc/frc*^	na	205.67 ± 10.09
7584	P7	70	mEMS4922	*Nr2e1* ^*frc/frc*^	na	376.15 ± 38.77
7582	P21	39	mEMS4914	*Nr2e1* ^*frc/frc*^	10	183.67 ± 2.33
7588	P21	42	mEMS4914	*Nr2e1* ^*frc/frc*^	4	197.67 ± 5.61
7599	P21	45	mEMS4922	*Nr2e1* ^*frc/frc*^	9	146.33 ± 5.78
7597	P21	54	mEMS4922	*Nr2e1* ^*frc/frc*^	nd	160.00 ± 0.00
7587	P21	61	mEMS4914	*Nr2e1* ^*frc/frc*^	7	170.00 ± 7.64
7583	P21	64	mEMS4914	*Nr2e1* ^*frc/frc*^	1	160.00 ± 5.77
7600	P21	66	mEMS4922	*Nr2e1* ^*frc/frc*^	5	149.33 ± 4.70
7604	P21	67	mEMS4922	*Nr2e1* ^*frc/frc*^	5	127.50 ± 7.50
7589	P21	77	mEMS4914	*Nr2e1* ^*frc/frc*^	1	Irregular morphology
7598	P21	86	mEMS4922	*Nr2e1* ^*frc/frc*^	7	108.33 ± 4.81
7578	P7	29	mEMS4919	*Nr2e1* ^*+/+*^	na	204.43 ± 18.80
7580	P7	46	mEMS4919	*Nr2e1* ^*+/+*^	na	259.95 ± 42.37
7579	P7	51	mEMS4919	*Nr2e1* ^*+/+*^	na	196.42 ± 4.99
7581	P7	71	mEMS4919	*Nr2e1* ^*+/+*^	na	180.5 ± 8.59
7612	P21	0.3	mEMS4926	*Nr2e1* ^*+/+*^	11	185.00 ± 4.93
7611	P21	10	mEMS4926	*Nr2e1* ^*+/+*^	11	190.33 ± 14.90
7610	P21	11	mEMS4926	*Nr2e1* ^*+/+*^	11	159.33 ± 6.33
7593	P21	18	mEMS4919	*Nr2e1* ^*+/+*^	11	178.00 ± 8.00
7592	P21	45	mEMS4919	*Nr2e1* ^*+/+*^	10	193.67 ± 5.84
7595	P21	50	mEMS4919	*Nr2e1* ^*+/+*^	13	172.67 ± 11.85
7591	P21	54	mEMS4919	*Nr2e1* ^*+/+*^	11	Irregular morphology
7594	P21	58	mEMS4919	*Nr2e1* ^*+/+*^	10	190 ± 0.00
7590	P21	92	mEMS4919	*Nr2e1* ^*+/+*^	9	204.33 ± 7.17

na, not applicable; nd, not done

**Figure 1:**
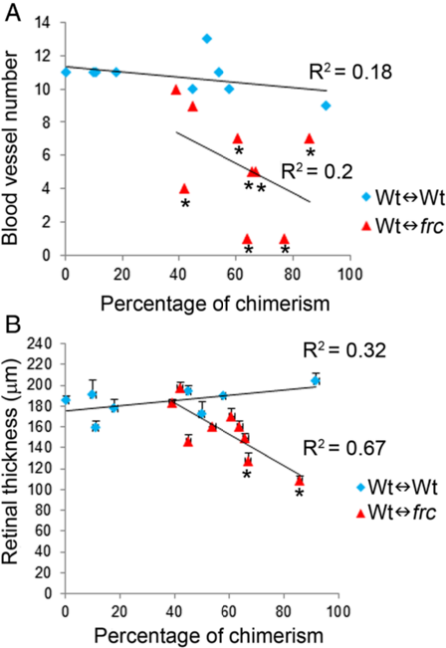
Reduced retinal thickness and blood vessel numbers of *Nr2e1* ^*frc/frc*^ retinas were rescued in Wt↔*frc* chimeras. **a** The blood vessel number of each chimera was assessed by funduscopy. Scatter plot showed normal blood vessel numbers in chimeric retinas containing up to 45 % *Nr2e1* ^*frc/frc*^ cells. *n* = 9 for Wt↔Wt, *n* = 9 for Wt↔*frc*. **b** Retinal thickness in each chimera was measured in 3 sections; a central section containing the optic nerve, and two sections 60 μm away in both directions. Scatter plot showed normal retinal thickness in chimeric retinas containing up to 66 % *Nr2e1* ^*frc/frc*^ cells. *n* = 8 for Wt↔Wt, *n* = 9 for Wt↔*frc*; *, Z-score ≤ −3; error bars represent SEM

We took fundus images of the chimeric eyes and manually counted the numbers of blood vessels in each chimera. We found a very poor correlation (R^2^ = 0.2) between the numbers of blood vessels and the percentage of chimerism in Wt↔*frc* eyes (Fig. 1a). Interestingly, only two chimeric eyes with the lowest percentage of chimerism (39 % and 45 %) had normal blood vessel numbers (10 and 9 blood vessels, respectively; Z-score higher than −3), suggesting that a contribution ≥55 % wild-type cells was necessary to rescue normal blood vessel development (Fig. 1a).

We measured the retinal thickness in a central section containing the optic nerve and two other adjacent sections 60 μm away in both directions. We found that the percentage of chimerism did not affect the retinal thickness of Wt↔Wt chimeras (Fig. 1b) but it affected the thickness of Wt↔frc chimeras (R^2^ = 0.67). In this case, a 66 % chimeric retina had a retinal thickness comparable to wild type (146.33 ± 5.78 μm, Z-score greater than −3) suggesting that ≥34 % wild-type cells were needed to rescue it (Fig. 1b). Only the two highest percentage chimeric retinas (67 % and 86 %) were not rescued by wild-type cells having thinner than wild-type retinas (127.5 ± 7.5 and 108.33 ± 4.81 μm, respectively, Z-score lower than −3).

In summary, a high contribution of wild-type cells (≥55 %) was needed to achieve rescue of blood vessels whereas retinal thickness was restored by fewer wild-type cells (≥34 %). The rescue of retinal thickness suggests that cell loss is not regulated cell autonomously by Nr2e1. We then studied whether other retinal abnormalities occurring in *Nr2e1* null mice including gliosis and gross structural defects could also be rescued by wild-type cells in chimeric retinas.

### *Nr2e1*^*frc/frc*^ Müller glia misexpression of GFAP and retinal structural defects were rescued in Wt↔*frc* chimeras

**Figure 2:**
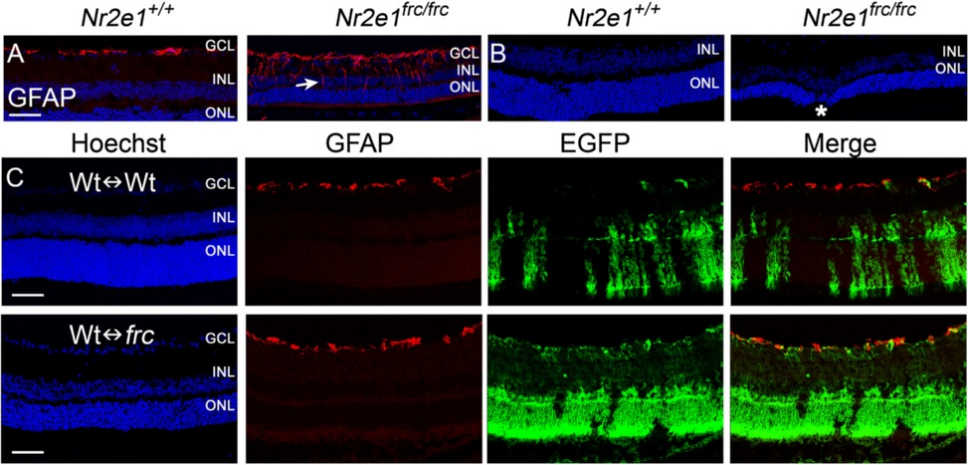
Nr2e1^frc/frc^-Müller-glia misexpression of GFAP and retinal structural defects were rescued in Wt↔*frc* chimeras. Transverse retinal sections from P21 mice were immunostained for GFAP (red). **a** GFAP was observed in the GCL of wild-type and *Nr2e1* ^*frc/frc*^ retinas but also in the processes of Müller glia in *Nr2e1* ^*frc/frc*^ retinas (arrow). **b** *Nr2e1* ^*frc/frc*^ retinas had abnormal INL intrusions into the ONL (asterisk). **c** A Wt↔*frc* chimera with 86 % *Nr2e1* ^*frc/frc*^ cells (EGFP positive, green) showed absence of GFAP expression in Müller cells and absence of INL intrusions into the ONL. GCL, ganglion cell layer; INL, inner nuclear layer; ONL, outer nuclear layer; Hoechst, nuclear counterstain (blue); scale bar = 50 μm. *n* = 9 for Wt↔Wt, *n* = 10 for Wt↔*frc*

As previously reported [21], we found that central Müller glia of *Nr2e1*^*frc/frc*^ retinas express GFAP (Fig. 2a), a neurofilament protein that is normally expressed only in astrocytes and reactive Müller glia [24]. We also observed that, in addition to its thinner size, *Nr2e1*^*frc/frc*^ retinas present structural defects including regions of INL and ONL that overlap with an apparent loss of the OPL (Fig. 2b). We observed that these defects occurred 2.43 ± 1.36 times per mm in the mutant retinas.

We found that none of the chimeric retinas, even those with a very high contribution of *Nr2e1*^*frc/frc*^ cells, express GFAP in central Müller glia, suggesting that wild-type cells rescue this defect (Fig. 2c). We also observed that the retina of high percentage chimeras was still very thin but did not have the structural defects seen in *Nr2e1*^*frc/frc*^ retinas (Fig. 2c).

In summary, misexpression of GFAP and gross structural defects do not emerge cell-autonomously in *Nr2e1*^*frc/frc*^ retinas and are likely a consequence of other defects such as abnormal cell type development. We then focused on studying the numbers of each retinal cell type in P7 *Nr2e1*^*frc/frc*^ and chimeric retinas.

### Lack of *Nr2e1* resulted in increased numbers of glycinergic amacrine cells and S-cones, and normal numbers of Müller glia

Although the role of Nr2e1 in regulating retinal thickness has been assessed [21, 22], the numbers of different retinal cell types in *Nr2e1*-null retinas has not yet been studied. Similarly, the presence of each retinal cell type has previously only been assessed in adult eyes after apoptosis has already severely altered the structure and composition of the retina [21, 22]. To gain better insight into the cell numbers generated in *Nr2e1*^*frc/frc*^ mouse retinas, we studied the retina at P7. At this time point most cells have already been differentiated and less than 5 % of bipolar and Müller glia are still being generated in the rat retina [25]. At P7, cells have not been exposed to daily visual activity and are less prone to apoptosis in *Nr2e1* null retinas [22]. To quantitatively assess differences in cell numbers, we manually counted all cell types in the retinas of wild-type and *Nr2e1*^*frc/frc*^ mice at P7. Five sections throughout the eyes were analyzed.

To better understand the interplay between wild-type and *Nr2e1*^*frc/frc*^ cells during the development of chimeric retinas, and to better assess clonal composition, we looked at the density of different retinal cell types belonging to each genotype in Wt↔*frc* chimeras at P7. The number of each EGFP positive or EGFP negative cell type was divided over the EGFP-positive or EGFP-negative area, respectively.

Previous studies have shown a reduced outer nuclear layer (ONL) but have conflicting results regarding the thickness of the inner nuclear layer (INL) in P4-P7 *Nr2e1* mutant retinas [21, 22]. Miyawaki et al., reported a thicker INL [21] whereas Zhang et al., reported a thinner INL [22]. We observed, in agreement with a reduced ONL, a 33 % reduction in rods in *Nr2e1*^*frc/frc*^ retinas when compared to wild type (Additional file 2: Figure S2A and C). We found no difference in INL thickness between wild-type and *Nr2e1*^*frc/frc*^ P7 central retinas measured in sections taken through the optic nerve (Wt = 85.9 ± 0.17; frc = 85.86 ± 3.27; *n* = 3 for Wt; *n* = 3 for frc).

We also found a 27 % reduction in bipolar cells in *Nr2e1*^*frc/frc*^ retinas when compared to wild type (Additional file 2: Figure S2B and C). A reduction in the numbers of rods and bipolar cells is expected given that they differentiate at later time-points in the mouse retina [26]. However, since the thickness of the *Nr2e1*^*frc/frc*^ INL is not overall reduced at P7, other cell types must be overrepresented in these retinas.

In Wt↔*frc* chimeras, *Nr2e1*^*frc/frc*^ rods (Additional file 2: Figure S2D and F) and bipolar cells (Additional file 2: Figure S2E and G) were 50 % and 53 % less abundant, respectively, compared to wild type. This suggests that wild-type cells cannot rescue the numbers of *Nr2e1*^*frc/frc*^ rods and bipolar cells that fail to differentiate.

**Figure 3:**
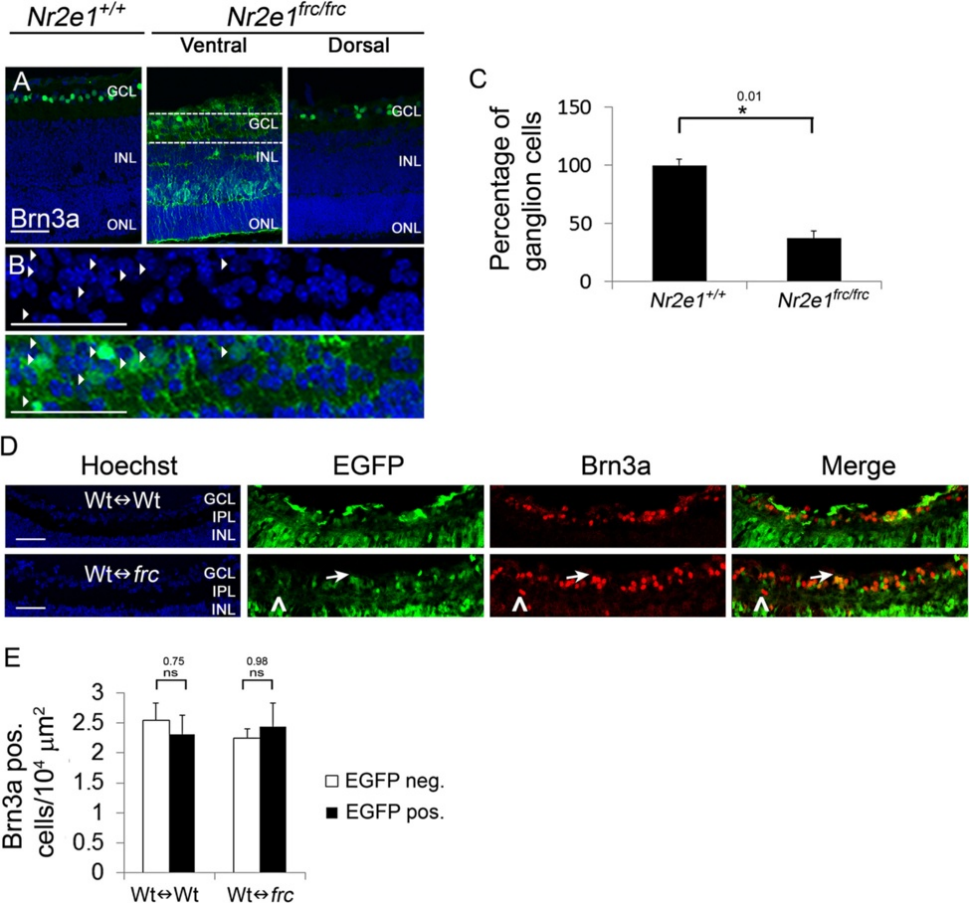
*Nr2e1* ^*frc/frc*^ P7 retinas had reduced numbers of ganglion cells that were rescued in Wt↔*frc* chimeras. Transverse retinal sections from P7 *Nr2e1* ^*+/+*^, *Nr2e1* ^*frc/frc*^, and chimeric mice were immunostained for Brn3a (ganglion cells). **a** *Nr2e1* ^*frc/frc*^ retinas had less Brn3a positive cells (green)in the GCL and misexpressed Brn3a in cells ofthe ventral INL that resembled Müller glia **b** Magnification of the rectangle in A showing Brn3anuclear staining in some GCL cell nuclei (arrow heads) and cytoplasmic projectionsresembling Müller glia end-feet **c** Ganglion cells were counted throughout five sections across the retina of *Nr2e1* ^*+/+*^ and *Nr2e1* ^*frc/frc*^ mice. Numbers were normalized to retinal length and expressed as percentages of *Nr2e1* ^*+/+*^ cell numbers. Reduced numbers of ganglion cells were observed in *Nr2e1*-mutant retinas compared to wild type (63 % decrease). **d** In Wt↔Wt and Wt↔*frc* chimeras, the density of EGFP positive (green) ganglion cells (arrows), appeared similar to the density of EGFP negative ganglion cells. Representative images of a 71 % Wt↔Wt and a 58 % Wt↔*frc* chimeric retina are shown. Brn3a positive cells were mislocalized in the IPL (arrowheads) and INL of Wt↔*frc* retinas. **e** Quantification of the density of ganglion cells that were derived from host blastocyst or ESCs in chimeras was assessed by counting single-labeled cells (Brn3a positive and EGFP negative) or double-labeled cells (Brn3a positive and EGFP positive) and dividing them by the EGFP negative or EGFP positive retinal area (ONL + INL), respectively. The density of *Nr2e1* ^*frc/frc*^ ganglion cells (EGFP positive) was similar to the density of wild-type ganglion cells (EGFP negative) in Wt↔*frc* chimeras. *n* = 3 for *Nr2e1* ^*+/+*^, *n* = 3 for *Nr2e1* ^*frc/frc*^, *n* = 3 for Wt↔Wt, *n* = 3 for Wt↔*frc*;*, P ≤ 0.05; ns, not significant; error bars represent SEM. GCL, ganglion cell layer; Hoechst, nuclear counterstain (blue); INL, inner nuclear layer; neg., negative; ONL, outer nuclear layer; pos., positive; scale bar = 50 μm

We also observed a reduction of Brn3a-positive ganglion cells in *Nr2e1*^*frc/frc*^ retinas, concomitantly with an ectopic expression of this marker in the ventral retina (Fig. 3a). This ectopic expression was seen in cell somas located in the INL and in cytoplasmic projections with termini that resemble Müller glia end-feet in the GCL (Fig. 3a and b). Müller glia end-feet normally have a cobblestone pattern and enclose the ganglion cell soma [27]. Quantification of cell numbers in the GCL revealed that *Nr2e1*^*frc/frc*^ retinas have a 63 % decrease in Brn3a positive ganglion cells (Fig. 3c). However, this lower numbers were rescued in Wt↔*frc* chimeric retinas (Fig. 3d and e). Furthermore, we also observed many Brn3a positive cells that aberrantly localized in the IPL and adjacent INL of chimeric retinas (Fig. 3d).

**Figure 4:**
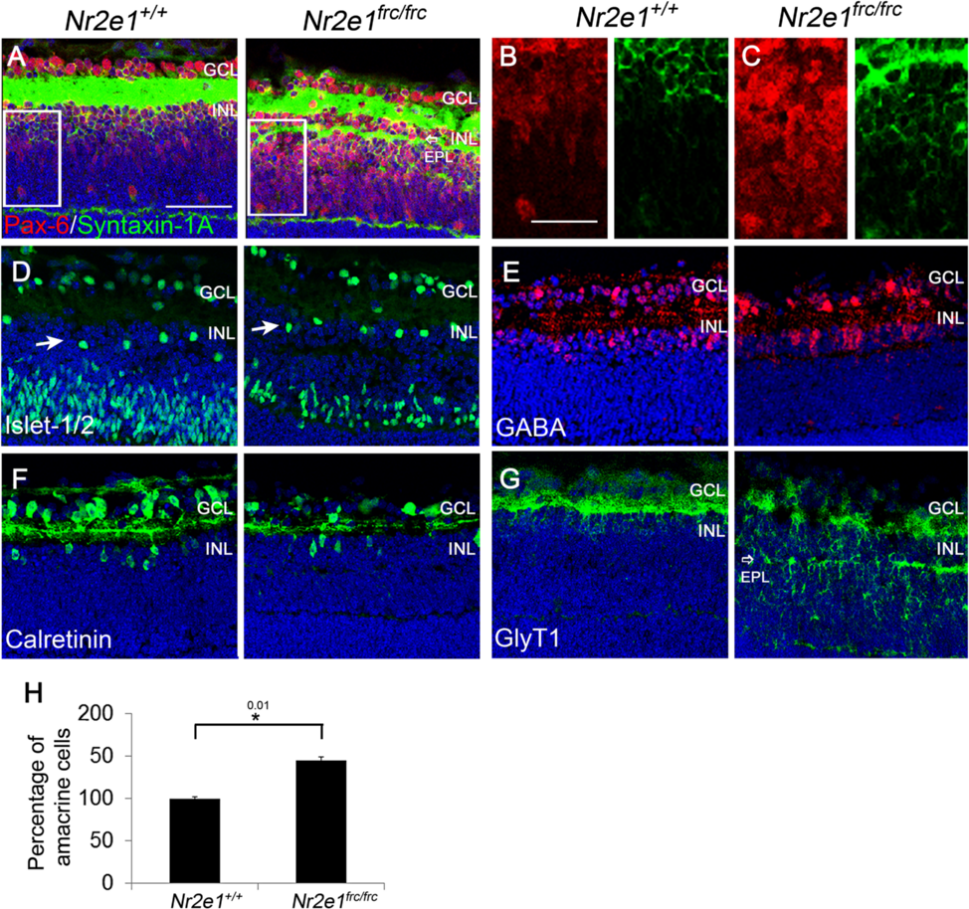
*Nr2e1* ^*frc/frc*^ P7 retinas had increased numbers of glycinergic amacrine cells. Transverse retinal sections from P7 *Nr2e1* ^*+/+*^ and *Nr2e1* ^*frc/frc*^ mice were immunostained for the pan-amacrine markers Pax-6 and syntaxin-1A, and the subclass markers Islet-1/2, GABA, calretinin, and glycine transporter 1 (GlyT1). **a** Retinal section showing amacrine cells immunostained with Pax-6 (red) and syntaxin-1A (green). *Nr2e1* ^*frc/frc*^ retinas had more Pax-6 and syntaxin-1A positive cells than wild-type retinas, and these cells occupied the majority of the INL. An ectopic plexiform layer (EPL) is also indicated (open arrow). Magnification of the Pax-6 (red) and syntaxin-1A (green) staining is shown for a region of **b** *Nr2e1* ^*+/+*^ or **c** *Nr2e1* ^*frc/frc*^ retina (rectangle). **d**-**f** Neither (**d**) Islet-1/2 positive (green) (arrows), (**e**) GABA positive (red), or (**f**) calretinin positive amacrine cells appeared increased in *Nr2e1*-mutant retinas. **g** In contrast, GlyT1 positive amacrine cells appeared increased in the INL of *Nr2e1*-mutant retinas. EPL (open arrow). **h** Amacrine cells were counted in the GCL throughout five sections across the retina of *Nr2e1* ^*+/+*^ and *Nr2e1* ^*frc/frc*^ mice. Numbers were normalized to retinal length and expressed as percentages of *Nr2e1* ^*+/+*^ cell numbers. Increased numbers of amacrine cells (assessed as Pax6 positive minus Brn3a positive) were observed in the GCL of *Nr2e1*-mutant retinas compared to wild type (45 % increase). *n* = 3 for *Nr2e1* ^*+/+*^, *n* = 3 for *Nr2e1* ^*frc/frc*^; *, P ≤ 0.05; error bars represent SEM. EPL, ectopic plexiform layer; GCL, ganglion cell layer; Hoechst, nuclear counterstain (blue); INL, inner nuclear layer; scale bar = 50 μm in all images except for B where it represents 12.5 μm

In contrast to the reduction in rods, bipolar, and ganglion cells, Pax-6 and syntaxin-1A positive amacrine cells were increased in numbers in the INL of *Nr2e1*^*frc/frc*^ retinas, representing the majority of cells in this layer (Fig. 4a and b). Quantification of amacrine cells in the GCL (assessed as Pax-6 positive minus Brn3a positive cells) revealed a 45 % increase in mutant amacrine cells compared to wild type (Fig. 4h). To better characterize these cells, we stained the retina with antibodies for proteins expressed in subpopulations of amacrine cells. Interestingly, the excess Pax-6 and syntaxin-1A positive cells were negative for the GABAergic amacrine-cell markers GABA, calretinin, and Islet-1/2 but positive for glycine transporter 1 (GlyT1), a marker of glycinergic amacrines (Fig. 4d-g). This suggests that *Nr2e1*^*frc/frc*^ retinas generated preferentially an excess of glycinergic amacrine cells.

**Figure 5:**
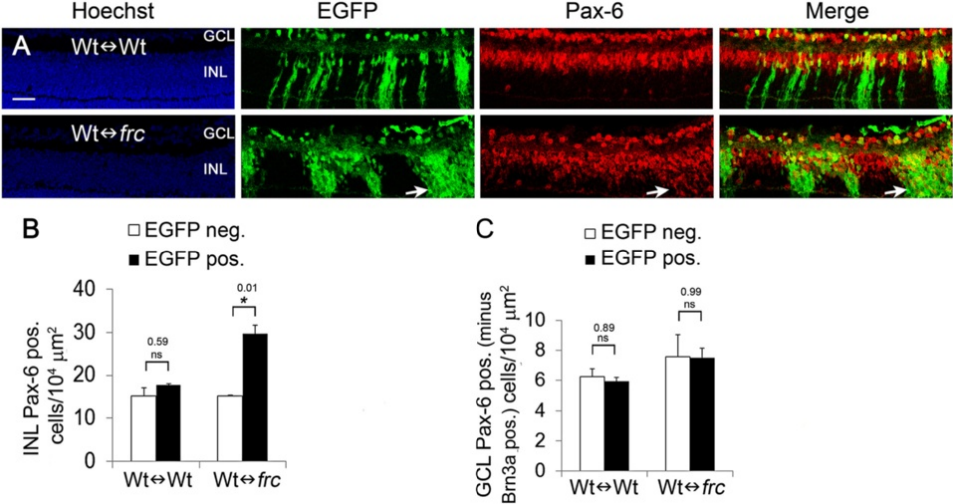
Increased numbers of *Nr2e1* ^*frc/frc*^ amacrine cells were not rescued in P7 Wt↔*frc* chimeras. Chimeric eyes were immunostained for the pan-amacrine marker Pax-6. **a** In Wt↔Wt chimeras, the density of EGFP positive (green) amacrine cells appeared similar to the density of EGFP negative amacrine cells. In Wt↔*frc* chimeras, the density of amacrine cells that were EGFP positive (green) appeared higher than the density of EGFP negative amacrine cells. Representative images of a 29 % Wt↔Wt and a 58 % Wt↔*frc* chimeric retina are shown. The arrow shows a region with high numbers of mutant cells (EGFP positive) where amacrine cells (Pax-6 positive) are overrepresented. **b,c** Quantification of the density of amacrine cells that were derived from host blastocyst or ESCs in chimeras was assessed by counting single-labeled cells (Pax-6 positive and EGFP negative) or double-labeled cells (Pax-6 positive and EGFP positive) and dividing them by the EGFP negative or EGFP positive retinal area (ONL + INL), respectively. Cell counts were performed in both the INL and the GCL. In the INL, large and round nuclei adjacent to the OPL, presumably belonging to horizontal cells, were excluded from counting. Amacrine numbers in the GCL were obtained by subtracting Brn3a positive ganglion cells from the total Pax-6 positive cells in this layer. In Wt↔*frc* chimeras, the density of *Nr2e1* ^*frc/frc*^ amacrine cells (EGFP positive) was (**b**) higher (49 % increase) than the density of wild-type amacrine cells in the INL but was (**c**) similar to wild type in the GCL. *n* = 3 for Wt↔Wt, *n* = 3 for Wt↔*frc*; *, P ≤ 0.05; ns, not significant; error bars represent SEM; GCL, ganglion cell layer; Hoechst, nuclear counterstain (blue); INL, inner nuclear layer; neg., negative; pos., positive; scale bar = 50 μm

In the INL of Wt↔*frc* chimeric retinas, *Nr2e1*^*frc/frc*^ amacrine cells were 49 % more abundant than wildtype (Fig. 5a and b). In agreement with this increase, there was an overrepresentation of mutant GLYT1 positive cells in chimeras (Additional file 3: Figure S3). In contrast, the numbers of amacrine cells in the GCL of Wt↔*frc* chimeric retinas were comparable to wild type (Fig. 5c), suggesting that wild-type cells prevented the migration or survival of excess amacrine cells in the GCL.

**Figure 6:**
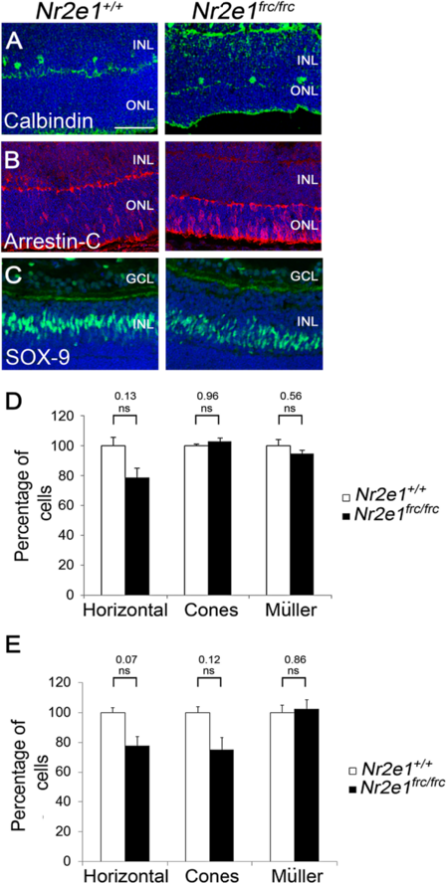
*Nr2e1* ^*frc/frc*^ retinas had normal numbers of horizontal, cone, and Müller glia cells. Transverse retinal sections from P7 and P21 *Nr2e1* ^*+/+*^ and *Nr2e1* ^*frc/frc*^ mice were immunostained for calbindin (horizontal cells, adjacent to the ONL), arrestin (cones), and SOX-9 (Müller glia). **a**-**c** P7 retinal sections showing (**a**) horizontal cells in green, (**b**) cones in red, and (**c**) Müller glia in green. **d**,**e** Each retinal cell type was counted throughout five sections across the retina of (**d**) P7 or (**e**) P21 *Nr2e1* ^*+/+*^ and *Nr2e1* ^*frc/frc*^ mice. Numbers were normalized to retinal length and expressed as percentages of *Nr2e1* ^*+/+*^ cell numbers. Normal numbers of horizontal, cone, and Müller glia cells were observed in *Nr2e1*-mutant retinas compared to wild type at both time-points. *n* = 3 for *Nr2e1* ^*+/+*^, *n* = 3 for *Nr2e1* ^*frc/frc*^; *, P ≤ 0.05; ns, not significant; error bars represent SEM. GCL, ganglion cell layer; INL, inner nuclear layer; ONL, outer nuclear layer; scale bar = 50 μm

In P7 *Nr2e1*^*frc/frc*^ retinas, the densities of horizontal, cones, and Müller glial cells were similar to wild-type (Fig. 6a-d). Interestingly, these densities were also normal at P21 (Fig. 6e) suggesting that horizontal, cones, and Müller glial cells were spared from the excessive apoptosis that occurs during the postnatal period in *Nr2e1*^*frc/frc*^ retinas [22].

**Figure 7:**
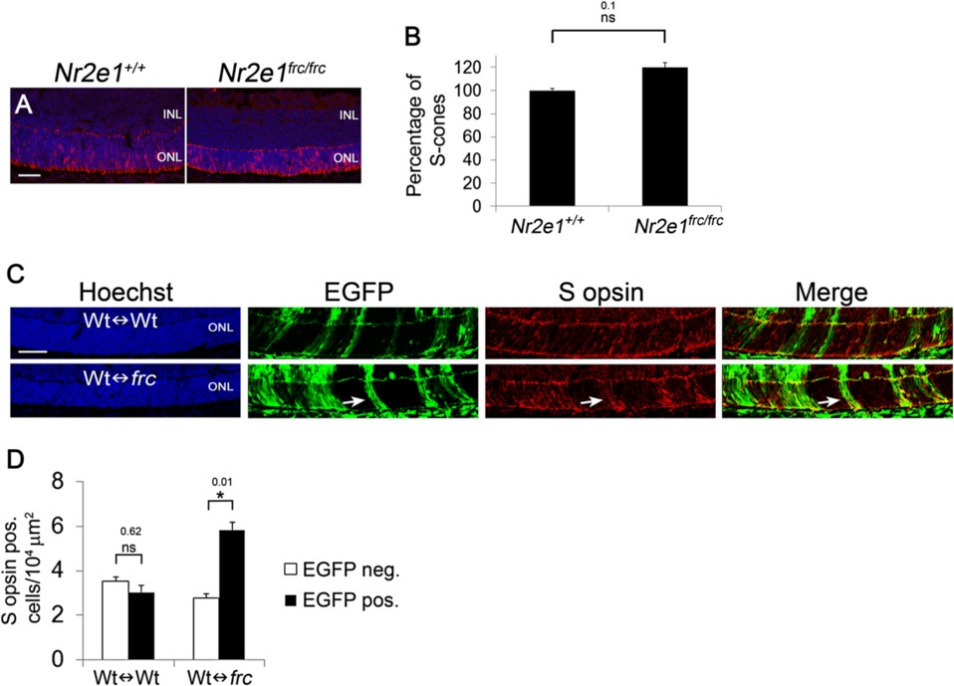
*Nr2e1* ^*frc/frc*^ S-cones were overrepresented in Wt↔*frc* chimeras. Transverse retinal sections from P7 *Nr2e1* ^*+/+*^, *Nr2e1* ^*frc/frc*^, and chimeric mice were immunostained for S opsin (S-cones). **a** Retinal sections showing S-cones labeled with S Opsin (red). **b** To assess differences in S-cones numbers between wild-type and *Nr2e1*-mutant retinas, S opsin positive cells were counted throughout five sections across the retina of P7 *Nr2e1* ^*+/+*^ and *Nr2e1* ^*frc/frc*^ mice. Numbers were normalized to retinal length and expressed as percentages of *Nr2e1* ^*+/+*^ cell numbers. There was no significant difference between the numbers of S opsin positive cells in wild-type and *Nr2e1*-mutant retinas. **c** Retinal sections of chimeras showing S opsin in red. The density of *Nr2e1* ^*frc/frc*^ S opsin positive cells appeared higher in *Nr2e1*-mutant regions (EGFP positive) compared to wild-type regions. The arrows show a region with *Nr2e1*-mutant cells and a high density of S-cones. Representative images of a 50 % Wt↔Wt and a 39 % Wt↔*frc* chimeric retina are shown. **d** Quantification of the density of S-cone cells that were derived from host blastocyst or ESCs in chimeras was assessed by counting single-labeled cells (S opsin positive and EGFP negative) or double-labeled cells (S opsin positive and EGFP positive) and dividing them by the EGFP negative or EGFP positive retinal area (ONL + INL), respectively. In Wt↔Wt chimeras, the density of EGFP positive and EGFP negative S-cone cells was similar while in Wt↔*frc* chimeras the density of *Nr2e1* ^*frc/frc*^ S-cone cells (EGFP positive) was higher (48 % increase) than the density of wild-type S-cone cells (EGFP negative). *n* = 3 for *Nr2e1* ^*+/+*^, *n* = 3 for *Nr2e1* ^*frc/frc*^, *n* = 3 for Wt↔Wt, *n* = 3 for Wt↔*frc*; *, P ≤ 0.05; neg., negative;ns, not significant; error bars represent SEM. GCL, ganglion cell layer; Hoechst, nuclear counterstain (blue); INL, inner nuclear layer; ONL, outer nuclear layer; pos., positive; scale bar = 50 μm

Similarly, there was no difference in the number of S-cones labeled with S opsin between P7 *Nr2e1*^*frc/frc*^ and *Nr2e1*^*+/+*^ retinas (Fig. 7a and b). However, in P7 Wt↔*frc* chimeras the difference between *Nr2e1*^*frc/frc*^ and *Nr2e1*^*+/+*^ S-cone numbers was very obvious (Fig. 7c, arrow). Quantification of S Opsin positive cells in P7 Wt↔*frc* revealed a 48 % increase in *Nr2e1*^*frc/frc*^ S-cones compared to wild type (Fig. 7c and d).

In summary, P7 *Nr2e1*^*frc/frc*^ retinas have a marked reduction of ganglion, bipolar, and rod cells, increased numbers of glycinergic amacrine cells, and normal numbers of cones, horizontal, and Müller glial cells. In Wt↔*frc* P7 chimeric retinas, an increase in *Nr2e1*^*frc/frc*^ S-cone numbers was also evident. In the same chimeras, wild-type cells did not rescue *Nr2e1*^*frc/frc*^ abnormal cell numbers with the exception of ganglion and amacrine cells in the GCL. We further studied whether these cellular defects were accompanied by abnormalities in retinal lamination as explained below.

### *Nr2e1*^*frc/frc*^ retinas displayed an ectopic plexiform layer and a disorganized inner plexiform layer, which were not rescued by wild-type cells in Wt↔*frc* retinas

**Figure 8:**
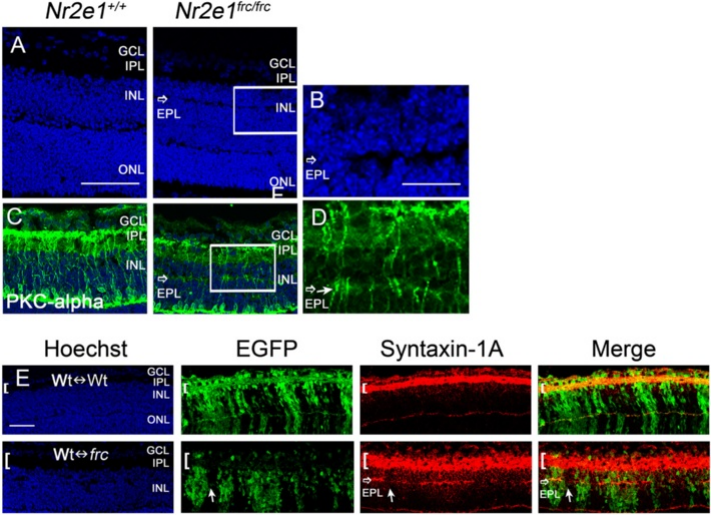
*Nr2e1* ^*frc/frc*^ retinas displayed an ectopic plexiform layer that was not rescued in Wt↔*frc* chimeras. Transverse retinal sections from P7 *Nr2e1* ^*+/+*^, *Nr2e1* ^*frc/frc*^, and chimeric mice were immunostained for PKC-alpha or syntaxin-1A. **a** A region devoid of Hoechst-stained nuclei indicated an ectopic plexiform layer (EPL) in *Nr2e1* ^*frc/frc*^ retinas (open arrow). **b** Magnification of the box in A showing the EPL (open arrow). **c** PKC-alpha positive bipolar cells (green) extended processes into the IPL in wild-type retinas but also to the EPL (open arrow) in *Nr2e1*-mutant retinas. **d** Magnification of the box in G showing bipolar cell processes (solid arrow) in the EPL (open arrow). **e** Chimeras showing the distribution of syntaxin-1A (red) in the INL. Representative images of a 51 % Wt↔Wt and a 58 % Wt↔*frc* chimeric retina are shown. Note that syntaxin-1A positive EPL (open arrow) was seen in Wt↔*frc* chimeras in regions enriched with *Nr2e1*-mutant cells (EGFP positive, green). The solid arrows show a region with predominantly wild-type cells (EGFP negative) where the EPL is absent. Also note that the IPL (brackets) from Wt↔*frc* retina is thicker and more disorganized compared to Wt↔Wt. *n* = 3 for *Nr2e1* ^*+/+*^, *n* = 3 for *Nr2e1* ^*frc/frc*^, *n* = 4 for Wt↔Wt, *n* = 4 for Wt↔*frc*; EPL, ectopic plexiform layer; GCL, ganglion cell layer; Hoechst, nuclear counterstain (blue); INL, inner nuclear layer; ONL, outer nuclear layer; OPL, outer plexiform layer; scale bar = 50 μm

We observed that *Nr2e1*^*frc/frc*^ retinas displayed an ectopic plexiform layer (EPL) in the inner nuclear layer (INL) evident at P7 (Fig. 8a and b). Looking back, we can see that the EPL was labeled with the amacrine markers syntaxin-1A and GlyT1 throughout the retina (Fig. 4a and *g*, open arrow).

To evaluate whether bipolar cells that normally establish synaptic connections with retinal ganglion cells (RGCs) and amacrine cells in the inner plexiform layer (IPL) [28] also had terminals in the EPL, we stained retinas for PKC-alpha expressed in ON bipolars. We found that PKC-alpha positive bipolar cells had mislocalized axon branchings in the EPL (Fig. 8c and d, solid arrow).

We then asked if the lamination defects generated by *Nr2e1* mutant cells could be rescued by extracellular signals from wild-type cells in Wt↔*frc* chimeras. At P7 the EPL was present in all four chimeras, and regions of wild-type cells failed to form this layer, showing that wild-type cells cannot rescue this lamination defect or be influenced by mutant cells to form this ectopic layer (Fig. 8e).

Furthermore, the IPL of Wt↔*frc* chimeras was thicker throughout the whole retina and less defined than that of Wt↔Wt chimeras suggesting that the development of the IPL is also affected by the lack of *Nr2e1* (Fig. 8e, brackets).

To better characterize the EPL, we stained *Nr2e1*^*frc/frc*^ P7 retinas with antibodies that normally recognize the inner plexiform layer (IPL) and found that unlike syntaxin-1A and GlyT1, calbindin and mGluR1 were occasionally but not always found in this ectopic layer (Additional file 4: Figure S4A-D).

Moreover, some calbindin and mGluR1 positive cells were observed in proximity to the EPL of *Nr2e1*^*frc/frc*^ retinas (Additional file 4: Figure S4B and D, asterisks) and Pax-6-positive cells were found between mGluR1 positive sublaminae (Additional file 4: Figure S4D, arrowhead) revealing lamination defects.

**Figure 9:**
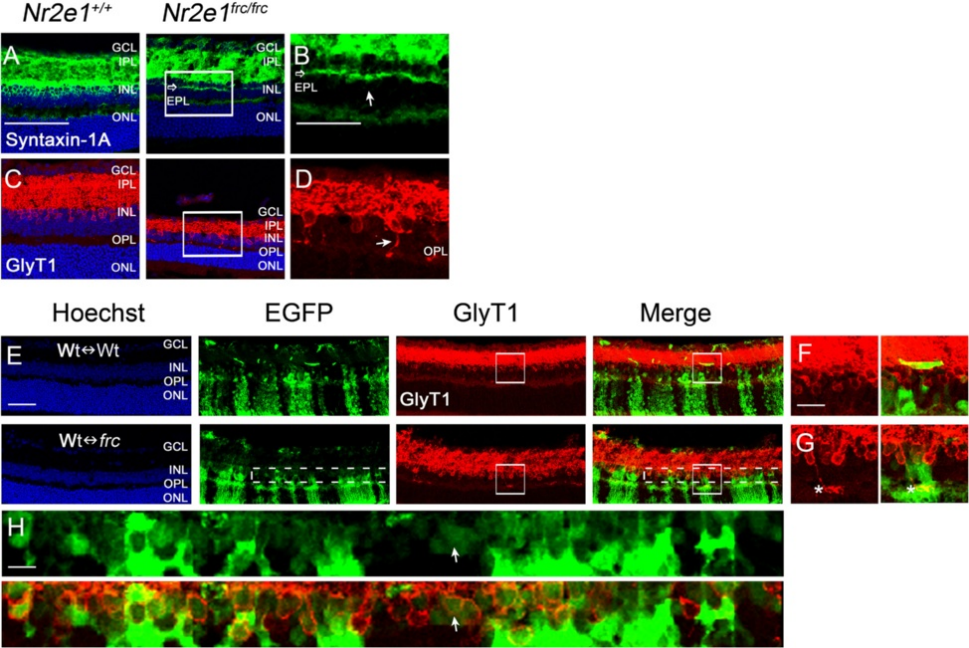
P21 retinas show lamination defects and loss of amacrine cells. Transverse retinal sections from P21 *Nr2e1* ^*+/+*^, *Nr2e1* ^*frc/frc*^, and chimeric mice were immunostained for syntaxin-1A or GlyT1. **a** In wild-type retinas, syntaxin-1A positive cells (green) were located in the INL with processes that projected into the IPL. In P21 *Nr2e1*-mutant retinas, syntaxin-1A positive processes projected to both the IPL and the EPL (open arrow). Note a decrease in syntaxin-1A positive cell bodies. **b** Magnification of the box in A showing the EPL (open arrow) of *Nr2e1*-mutant retinas positive for syntaxin-1A (solid arrow). **c** The glycinergic marker GlyT1 (red) revealed a reduction in the number of this subtype of amacrine cells in *Nr2e1* ^*frc/frc*^ retinas. **d** Magnification of the box in C showing the presence of many interplexiform amacrine cells extending neurites to the OPL in *Nr2e1* ^*frc/frc*^ retinas (solid arrow) indicative of additional lamination defects. In P21 chimeras, (**e**) the presence of many GlyT1 positive cells (red) in both Wt↔Wt and Wt↔*frc* is evident. Representative images of a 46 % Wt↔Wt and a 62 % Wt↔*frc* chimeric retina are shown. **f**,**g** Magnification of the boxes in E showing (**f**) not detectable interplexiform cells in the 46 % Wt↔Wt chimera and (**g**) the presence of strongly labeled interplexiform cells in the 62 % Wt↔*frc* chimera (asterisks). **h** Magnification of dotted boxes in E showing *Nr2e1*-mutant cells (EGFP positive, green) that are also GlyT1 positive (red) in the INL (exemplified by arrows). *n* = 3 for *Nr2e1* ^*+/+*^, *n* = 3 for *Nr2e1* ^*frc/frc*^, *n* = 3 for P21 Wt↔Wt, *n* = 3 for P21 Wt↔*frc*; EPL, ectopic plexiform layer; GCL, ganglion cell layer; Hoechst, nuclear counterstain (blue); INL, inner nuclear layer; ONL, outer nuclear layer; OPL, outer plexiform layer; scale bar in A to E = 50 μm; scale bar in F = 7 μm; scale bar in H = 9 μm

We also looked for the EPL in P21 retinas with antibodies against syntaxin-1A and GlyT1. We found that syntaxin-1A labeled the EPL in some regions of the retina despite extensive retinal dystrophy (Fig. 9a and b). We could not detect the EPL with GlyT1 but observed many GlyT1 positive interplexiform amacrine cells extending neurites towards the OPL in *Nr2e1*^*frc/frc*^ retinas (Fig. 9c and d). In P21 Wt↔*frc* chimeras there were many *Nr2e1*^*frc/frc*^ GlyT1 positive interplexiform amacrine cells suggesting that this phenotype is not corrected by wild-type cells (Fig. 9e-g).

Taken together, these results suggested a role of Nr2e1 in constraining the neurites of INL neurons to an organized IPL. P21 chimeric retinas also revealed a rescue of amacrine cell numbers by wild-type cells that we further studied.

### *Nr2e1*^*frc/frc*^ amacrine cell loss is rescued by wild-type cells in Wt↔*frc* retinas

We observed few syntaxin-1A and GlyT1 positive cells in P21 *Nr2e1*^*frc/frc*^ retinas suggesting extensive cell loss of amacrine cells as expected from dystrophy (Fig. 9a-d). However, in P21 Wt↔*frc* chimeras the numbers of *Nr2e1*^*frc/frc*^ GlyT1 positive amacrine cells appeared normal (Fig. 9h). Quantification of syntaxin-1A cell numbers in the INL of chimeras revealed normal numbers of *Nr2e1*^*frc/frc*^ amacrine cells (Additional file 5: Figure S5), suggesting that wild-type cells prevented the excessive loss of mutant amacrine cells during the postnatal period and that amacrine cell death is not a primary consequence of Nr2e1 loss.

The primary roles of Nr2e1 in the retina are then the regulation of RPC cell cycle, cell type numbersand inner neurite development. Nr2e1 remains to be expressed in the adult Müller glia and thus it may also have specific primary roles in this cell type. Therefore, we further characterized Müller glia with specific markers as shown below.

### *Nr2e1*^*frc/frc*^ Müller glia were aberrantly positioned in the inner nuclear layer and cell-autonomously misexpressed Brn3a

Nr2e1 is expressed in the Müller glia of adult mice [29]. To better characterize the expression of Nr2e1 in Müller glia during postnatal development, we stained P3, P7, P14, and P21 retinas with antibodies against β-gal and SOX-2 in mice expressing β-gal under the control of the human *NR2E1* promoter (*NR2E1-lacZ*) [29]. We made use of this mouse strain due to the lack of a reliable commercial antibody for Nr2e1. Previous work by others has shown that SOX-2 is expressed in progenitors and a subpopulation of amacrine cells starting at P0. By P7, SOX-2 expression becomes mainly restricted to the inner nuclear layer in a subpopulation of amacrine cells and in Müller glia. This expression in amacrine cells and Müller glia continues through adulthood [30]. We observed co-localization of SOX-2 and β-gal at all time-points in cells that extend apical and basal processes indicative of Müller glia (Additional file 6: Figure S6A). This suggests sustained expression of Nr2e1 in Müller glia throughout postnatal development. Furthermore, we observed SOX-2 positive cells in *Nr2e1*^*frc/frc*^ retinas at all time-points (Additional file 6: Figure S6B), further showing that *Nr2e1*^*frc/frc*^ Müller glia numbers are comparable to wild type.

**Figure 10:**
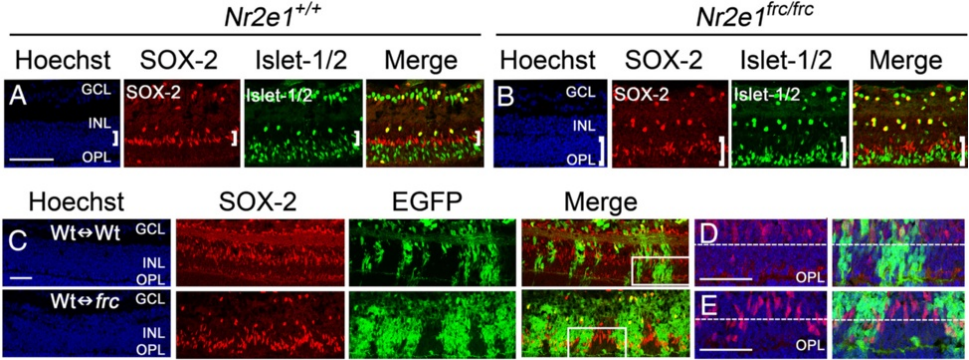
*Nr2e1* ^*frc/frc*^ Müller glia cells were aberrantly positioned in the INL**.** Transverse retinal sections from P7 *Nr2e1* ^*+/+*^, *Nr2e1* ^*frc/frc*^, and chimeric mice were immunostained for SOX-2 and Islet-1/2. **a** *Nr2e1* ^*+/+*^ SOX-2 positive Müller-glia somas (red, bracket) were localized between cholinergic amacrines in the lower INL and ON bipolars in the upper INL, the latter two cell types both labeled with Islet-1/2 (green). Müller-glia somas localized close to the middle of the INL and away from the OPL. **b** *Nr2e1* ^*frc/frc*^ Müller-glia somas (red, bracket) intermingled with ON bipolars (green) and localized adjacent to the OPL. **c** In Wt↔*frc* chimeras, the mispositioning of the Müller-glia soma was seen only in *Nr2e1*-mutant cells (EGFP positive, green), which were located closer to the OPL compared to wild-type cells (EGFP negative). Representative images of a 51 % Wt↔Wt and a 58 % Wt↔*frc* chimeric retina are shown. **d,e** Magnification of the boxes in (**c**) showing a region from the (**d**) Wt↔Wt chimera depicting Müller glia located close to the middle of the INL, and from the (**e**) Wt↔*frc* chimera depicting mutant Müller glia located adjacent to the OPL. The dotted lines represent an imaginary boundary above which most wild-type cells localize. Note that in a Wt↔*frc* chimera, most *Nr2e1*-mutant cells (EGFP positive) localize under this line close to the OPL and most wild-type cells (EGFP negative) localize above this line and away from the OPL. *n* = 3 for *Nr2e1* ^*+/+*^, *n* = 3 for *Nr2e1* ^*frc/frc*^, *n* = 4 for Wt↔Wt, *n* = 4 for Wt↔*frc*; EPL, Ectopic plexiform layer; GCL, ganglion cell layer; INL, inner nuclear layer; OPL, outer plexiform layer; Hoechst, nuclear counterstain (blue); scale bar = 50 μm

Several defects in the Müller glia of *Nr2e1*^*frc/frc*^ retinas were noticed suggesting a cell-autonomous role of Nr2e1 in these cells. For example, while wild-type Müller glia (SOX-2 positive) are positioned in the middle of the INL below bipolar cells, *Nr2e1*^*frc/frc*^ Müller glial somas are located adjacent to the OPL intermingling with bipolar cells (Fig. 10a-b). To ascertain whether this defect could be rescued by wild-type cells, we stained P7 chimeras for SOX-2. We found that, in Wt↔*frc* chimeras, the soma of *Nr2e1*^*frc/frc*^ Müller glia was still mislocalized whereas wild-type Müller glia localized correctly within the INL (Fig. 10c-e).

**Figure 11:**
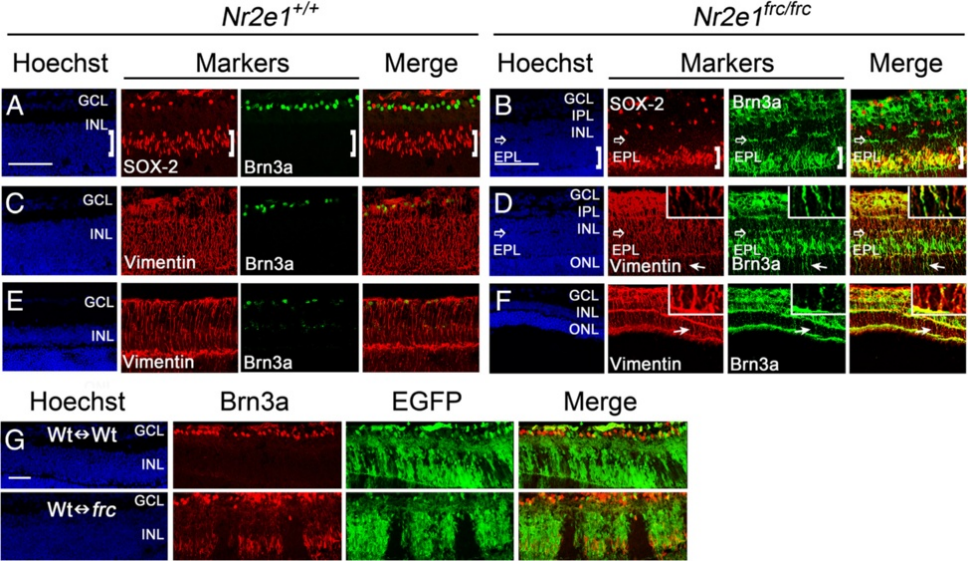
*Nr2e1* ^*frc/frc*^ Müller glia misexpressed Brn3a cell-autonomously**.** Transverse retinal sections from P7 and P21 *Nr2e1* ^*+/+*^ and *Nr2e1* ^*frc/frc*^ mice and P7 chimeras were immunostained for SOX-2, Brn3a, and Vimentin. **a** In P7 wild-type retinas, ganglion cells labeled with Brn3a (green) appeared as a different population from Müller glia labeled with SOX-2 (red, bracket). **b** In *Nr2e1* ^*frc/frc*^ retinas, the marker Brn3a (green) was misexpressed in Müller-glia somas (red, bracket), and in processes that branch in the EPL and IPL, with termini in the GCL that resemble end-feet. **c**-**f** Immunostaining for the Müller-glia markers vimentin (red) and Brn3a (green) in P7 and P21 retinas. In P7 retinas, these markers did not co-express in (**c**) *Nr2e1* ^*+/+*^ retinas but they did in (**d**) *Nr2e1* ^*frc/frc*^ retinas. This also occurred in P21 retinas where the markers did not co-express in (**e**) *Nr2e1* ^*+/+*^ retinas but they did in (**f**) *Nr2e1* ^*frc/frc*^ retinas. **d**,**f** Inset boxes show a magnified view of the vimentin and Brn3a co-localization in *Nr2e1* ^*frc/frc*^ Müller-glial processes. **g** In P7 Wt↔*frc* chimeras, misexpression of Brn3a (red) in Müller glia was seen in *Nr2e1*-mutant cells (EGFP positive, green) throughout the cell soma and processes, however, this was not seen in wild-type cells (EGFP negative) where Brn3a was only expressed in ganglion cells. Representative images of a 71 % Wt↔Wt and a 58 % Wt↔*frc* chimeric retina are shown. *n* = 3 for *Nr2e1* ^*+/+*^, *n* = 3 for *Nr2e1* ^*frc/frc*^, *n* = 4 for Wt↔Wt, *n* = 4 for Wt↔*frc*; EPL, Ectopic plexiform layer; GCL, ganglion cell layer; INL, inner nuclear layer; OPL, outer plexiform layer; Hoechst, nuclear counterstain (blue); scale bar = 50 μm

In addition, *Nr2e1*^*frc/frc*^ Müller glia misexpress the transcription factor Brn3a, which is normally expressed in sensory neurons including ganglion cells [31] (Fig. 11a and b). Interestingly, misexpression of this marker was only present in the ventral retina and was also mislocalized to the cell soma and processes instead of being restricted to the nuclear compartment (Fig. 11). The staining of Brn3a in the ventral retina appears diffuse and non-specific at first but careful examination suggested cytoplasmic expression in Müller glial projections, which span the whole retina and branch into the plexiform layers. To further confirm that the misexpression of Brn3a occurs in Müller glia, we also stained retinas for the Müller glia marker vimentin and observed its co-localization with Brn3a at both P7 and P21 (Fig. 11c-f and Additional file 7: Figure S7). Importantly, in P7 Wt↔*frc* chimeras the misexpression of Brn3a in Müller glia was evident in mutant but not in wild-type cells (Fig. 11g).

**Figure 12:**
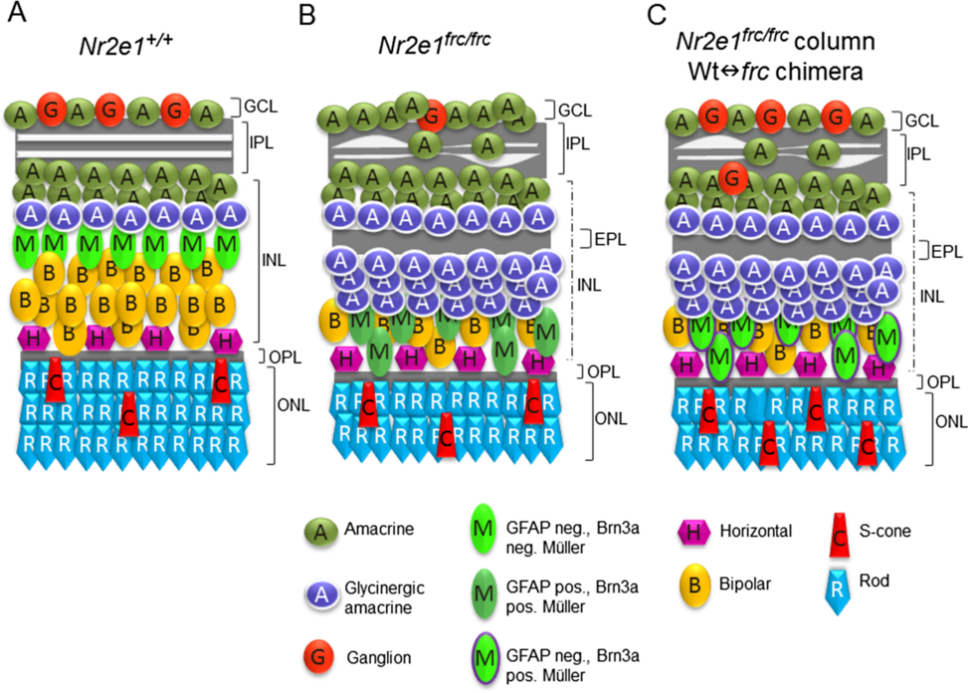
Model depicting the composition and organization of *Nr2e1* ^*+/+*^ and *Nr2e1* ^*frc/frc*^ retinal cells at P7. **a** Wild-type retina with 3 nuclear layers and 2 plexiform layers. **b** *Nr2e1* ^*frc/frc*^ retinas contain reduced numbers of ganglion, bipolar, and rod cells, but increased numbers of glycinergic amacrine cells. Disorganization of the IPL is evident, as well as the presence of an ectopic plexiform layer (EPL) in the INL. Müller glia misexpress GFAP and Brn3a, and have somas localized closer to the OPL intermingled with bipolar cells. **c** A predominantly *Nr2e1* ^*frc/frc*^ clone of cells in a Wt↔*frc* chimera has reduced numbers of bipolar and rods, but increased number of amacrine cells. Ganglion cell numbers are restored to wild type. There is also an increase in S-cone numbers compared to a wild-type clone. Disorganization of the IPL, and an EPL, are still evident. Müller glia misexpress Brn3a but not GFAP, and have mispositioned somas. Ganglion cells are ectopically positioned in the IPL and INL. EPL, ectopic plexiform layer; GCL, ganglion cell layer; INL, inner nuclear layer; IPL, inner plexiform layer; Neg., negative; ONL, outer nuclear layer; OPL, outer plexiform layer; Pos., positive

Overall, these results suggested that Nr2e1 regulates the maturaion of Müller glia cell-autonomously.

## Discussion

The phenotype of the *Nr2e1*-null eye is very complex involving various cell types and tissues, thus making the identification of the primary function of Nr2e1 during eye development challenging. In this study we used chimeras to clarify the cell-autonomous roles of Nr2e1 during retinogenesis, and described novel phenotypes of the *Nr2e1*^*frc/frc*^ mice in the P7 retina before extensive dystrophy has occurred. Our results suggest that Nr2e1 regulates the development of specific retinal cell-types and the organization of inner retinal neurites.

### Nr2e1 does not prevent retinal dystrophy cell-autonomously

The thickness of the P21-P28 *Nr2e1* mutant retina is highly reduced compared to wild type due to progressive postnatal cellular loss [21, 22]. Our findings suggest that wild-type cells can rescue cell loss in Wt↔*frc* chimeras. First, we showed that in P21 Wt↔*frc* chimeras, the reduced *Nr2e1*^*frc/frc*^ retinal thickness could be rescued by ≥34 % wild-type cells. Second, we observed that postnatal amacrine cell loss is rescued in P21 Wt↔*frc* chimeric retinas. These retinas appeared to have normal instead of reduced numbers of *Nr2e1*^*frc/frc*^ glycinergic amacrines and had normal numbers of *Nr2e1*^*frc/frc*^ total amacrine cells. Third, chimeras still had low numbers of mutant rods and bipolar cells that do not account for the observed rescue of retinal thickness. Fourth, we showed that low numbers of ganglion cells were rescued in P7 Wt↔*frc* chimeras. According to a previous study, increased apoptosis in the ganglion cell layer occurs in *Nr2e1* mutant retinas as early as P0 [21]. Thus, excessive cell-death may be the cause of ganglion cell reduction in P7 *Nr2e1*^*frc/frc*^ retinas that should be otherwise overrepresented as a consequence of premature neurogenesis. Therefore, the rescue of mutant ganglion cell numbers that we observed in chimeras is likely due to a restoration of ganglion cell survival. In conclusion, Nr2e1 does not have a cell-autonomous role in preventing postnatal cell death as wild-type cells are able to rescue cell loss.

Moreover, structural and gliosis-related phenotypes were rescued in P21 Wt↔*frc* chimeras. In *Nr2e1*^*frc/frc*^ retinas, some cells in the INL protrude into the ONL, and cells in the ONL migrate into the subretinal space [22]. In Wt↔*frc* chimeras, we do not see overlapping of INL and ONL cells or any other structural defect even in a retina with 86 % *Nr2e1*^*frc/frc*^ cells, suggesting that these defects are not regulated cell-autonomously by Nr2e1. Interestingly, expression of GFAP in Müller glia, which is typical during gliosis but also in *Nr2e1*^*frc/frc*^ retinas [21], was absent in *Nr2e1*^*frc/frc*^ Müller glia of chimeric retinas in spite of GFAP being a direct target of Nr2e1 [32]. This further supports the suggestion that dystrophy-related phenotypes are not regulated cell-autonomously by Nr2e1.

Furthermore, we observed that blood vessel numbers do not influence retinal thickness. *Nr2e1*^*frc/frc*^ reduced blood vessel number was rescued by ≥55 % wild-type cells. The high number of cells needed to rescue the blood vessel numbers indicates that this defect is not corrected by rescuing the mutant cells and is likely cell-autonomous. *Nr2e1* is expressed in proangiogenic astrocytes [20] and thus may act cell-autonomously in these cells to regulate retinal blood vessel development. In addition, the contribution of mutant cells to the astrocyte lineage could have been different than that to the retinal lineage, as astrocytes and retinal progenitors come from different cell populations. Regardless of the mechanism, it appears that the blood vessel and retinal thickness phenotypes of *Nr2e1*^*frc/frc*^ retinas are not influenced by each other and thus retinal dystrophy may not be influenced by abnormal blood vessel development. In addition, *Nr2e1*^*frc/frc*^ defects such as abnormal cellular proportions and lamination were never observed in wild-type regions of chimeric retinas suggesting that vascular abnormalities are not the primary cause of retinal defects in the mutant retinas.

These results taken together suggest that cell death, abnormal retinal structure, and gliosis, all related to dystrophy in *Nr2e1* mutant retinas, are not regulated cell-autonomously and can be rescued by wild-type cells. However, blood vessel abnormalities may not be a crucial factor in influencing dystrophy in *Nr2e1* mutant retinas. What are then the primary roles of Nr2e1 during retinogenesis? We found that in addition to the previously described role in cell-cycle regulation, Nr2e1 regulates the development of specific retinal cell types as discussed below.

### Nr2e1 regulates the development of specific retinal cell types

In the retina, Nr2e1 is thought to regulate cell numbers through shortening cell-cycle length and thus delaying neurogenesis [22]. We found that at P7, the density of some cell-types in *Nr2e1*^*frc/frc*^ retinas does not correspond to the proportions expected from premature cell-cycle exit. Importantly, the same cellular proportions were seen in *Nr2e1*^*frc/frc*^ regions of chimeras suggesting they emerge intrinsically from each clone of RPCs and are not secondary to eye volume differences.

We found that *Nr2e1*^*frc/frc*^ retinas are biased to generate Müller glia. The reduction in rods and bipolar cell numbers in *Nr2e1*^*frc/frc*^ retinascould be explained by underproduction of late-born cell types due to premature neurogenesis. Müller glia is the last retinal cell type to be generated [1] and thus premature neurogenesis would also predict reduced numbers. The fact that their numbers are not reduced in *Nr2e1*^*frc/frc*^ mice suggests a bias towards Müller glia differentiation in the absence of Nr2e1 regulation. In adult rat hippocampal-progenitors, Nr2e1 represses glial differentiation and activates the neuronal lineage [18]. Nr2e1 also directly binds to the promoter of glial-specific genes [32]. All of this taken together suggests a role for Nr2e1 in repressing the Müller glial lineage during development.

We also found an expanded population of glycinergic amacrine cells in the INL of *Nr2e1*^*frc/frc*^ retinas and increased numbers of *Nr2e1* mutant amacrine cells compared to wild type in chimeras. Amacrine cells are mainly subdivided in two groups: GABAergic and glycinergic [33]. Amacrine cell overproduction in *Nr2e1*^*frc/frc*^ retinas could be a consequence of precocious neurogenesis. However, a bias towards a particular subtype was not expected, especially when glycinergic amacrines are generated after the GABAergic subtype [33] making it more likely that GABAergic amacrines would be overrepresented. This suggests that Nr2e1 may have a role in regulating amacrine subtype development during mouse retinogenesis and that a switch in cell fate may have occurred in favor of glycinergic amacrine cells at the expense of other cell types that are reduced in number such as bipolar cells and rods.

We also observed that Nr2e1 regulates most retinal cell numbers cell-autonomously. To assess how Nr2e1 regulates extrinsic and intrinsic signals that affect RPC proliferation and differentiation in vivo, we evaluated the interplay between wild-type and *Nr2e1*^*frc/frc*^ cells in Wt↔*frc* chimeras. Intriguingly, the cellular composition of the GCL, but not the INL or ONL, was rescued by extra-cellular signals from wild-type cells. The GCL is comprised of ganglion and amacrine cells. The rescue of ganglion cell numbers in this layer is likely due to prevention of cell-death, as explained above. The fact that *Nr2e1*^*frc/frc*^ amacrine cell numbers were normal instead of overabundant in the GCL of chimeras suggests a role of extracellular signals in regulating their migration or survival in this layer rather than in preventing their generation because amacrines in the INL were still overabundant. Therefore, we think that wild-type cells did not affect the proportions of cells generated from RPCs. This suggests that RPC proliferation and differentiation does not depend on non-cell-autonomous roles of Nr2e1 as is the case of adult neural stem cell proliferation [19]. In other words, the role of Nr2e1 in differentiation and cell-cycle regulation is likely cell-autonomous in the retina.

Furthermore, chimeras revealed a role of Nr2e1 in S-cone development. In *Nr2e1*^*frc/frc*^ retinas, total cones and S-cones numbers were normal but in Wt↔*frc* chimeras *Nr2e1*^*frc/frc*^ S-cones were more abundant than wild-type S-cones. The late stage of development of S-cones, which includes S-Opsin expression, can be regulated by extracellular signaling such as the one mediated by thyroid hormone [34]. It is then possible that Nr2e1 regulates extrinsic signals necessary for the development and/or survival of S-cones. However, we cannot rule out that a bigger sample size would yield a significant difference in S-cones between wild-type and mutant retinas given that the quantified difference has a p-value of only 0.1. In agreement with this, a previous study reported increased S opsin transcript levels in P14 *Nr2e1* mutant retinas [22]. However, without information regarding protein levels and cell numbers, we cannot know with certainty that the numbers of S-cones were higher in those mutant retinas. Finally, the fact that Nr2e3, the closest relative of Nr2e1, regulates photoreceptor development and is mutated in enhanced S-cone syndrome in humans [35] further supports the hypothesis that Nr2e1 has a role in S-cone development.

Interestingly, in *Nr2e1*^*frc/frc*^ retinas, the densities of horizontal cells, cones, and Müller glia remained comparable to wild type from P7 through P21 when retinogenesis has long been completed. This indicates that these cells are spared from the excessive cell-death seen in *Nr2e1*^*frc/frc*^ retinas during the postnatal period. This also suggests that the majority of cell death observed between P7 and P21 in *Nr2e1*^*frc/frc*^ retinas may be due to the loss of amacrine cells that make most of the INL at P7.

Nr2e1 then has an important role in the development of certain cell-types during retinogenesis perhaps through the regulation of genetic networks that are also involved in cell-fate. During retinogenesis, transcription factors such as Prox1 [12], P57(Kip2) [14] and Math5 [15] are reused to perform multiple functions including cell proliferation and cell fate. Interestingly, Nr2e1 remains to be expressed in Müller glia but the specific roles of Nr2e1 in this cell type remains elusive. We further studied the expression of Nr2e1 during Müller glia development and the localization of Müller glia within the retina of *Nr2e1*^*frc/frc*^ and chimeric retinas as discussed below.

### Nr2e1 regulates Müller glia differentiation cell-autonomously

We observed that Nr2e1 is expressed in Müller glia throughout postnatal development and that *Nr2e1*^*frc/frc*^ Müller glia misexpress Brn3a and position their soma ectopically in the upper INL. Brn3a is a transcription factor normally expressed only in ganglion cell precursors and mature ganglion cells in the retina [36]. Brn3a ectopic expression was likely specific in Müller glia as 1) it was present only in the ventral retina; 2) it co-localized with Müller glial markers; and 3) it was present only in *Nr2e1*^*frc/frc*^ cells of chimeras. Thus, Nr2e1 may be required to maintain a differentiated state in Müller glia by directly or indirectly repressing Brn3a. Interestingly, Brn3a misexpression in Müller glia was also cytoplasmic and not exclusively nuclear. However, this is not surprising since various studies have reported cytoplasmic localization of other transcription factors including Crx [37], Hes [38], Pax-6, SOX-2 and Chx10 [39] in cultured Müller glia. This suggests that nuclear trafficking mechanisms of various transcription factors may work differently in Müller glia under atypical conditions. Importantly, Brn3a misexpression in the ventral retina suggests that Nr2e1 may have a role during dorso-ventral patterning in the retina as it does in the brain [40].

Both, the Brn3a misexpression and ectopic soma positioning of Müller glia were present only in *Nr2e1*^*frc/frc*^ cells of Wt↔*frc* chimeras. The fact that Nr2e1 is expressed in Müller glia and that wild-type Müller glia are unaffected by *Nr2e1*^*frc/frc*^ cells, suggests that Nr2e1 may have a cell-autonomous role in the maturation of Müller glia by regulating their transcriptional profile and localization within the retina. Furthermore, Müller glia mislocalization and misexpression of Brn3a are not a consequence of dystrophy since dystrophy is rescued by wild-type cells in chimeras but these Müller glia defects are not. *Nr2e1* null Müller glia were shown by others to have thinner processes compared to wild-type [21]. Our results suggest that this phenotype is likely cell-autonomous and not a consequence of other cellular defects in *Nr2e1* mutant retinas. However, this was not determined in that study.

The role of Nr2e1 in regulating the function of Müller glia or any other cell-type was not assessed in this study and deserves further investigation. However, defects in neuropil development were observed in *Nr2e1* mutant retinas, which suggest major defects in retinal connectivity and are discussed below.

### Nr2e1 regulates retinal lamination by preventing disorganization of inner retinal neurites

*Nr2e1*^*frc/frc*^ retinas have a disorganized IPL, an ectopic plexiform layer (EPL) within the INL, and abundant interplexiform amacrine cells. Together these observations suggest that Nr2e1 is involved in a cellular mechanism that organizes and constrains the neurites of inner retinal neurons to the IPL. Interestingly, the EPL can attract normal pre-synaptic partners, as bipolar cells arborized within it.

In Wt↔*frc* chimeras, wild-type regions did not have an EPL, however an EPL was evident in mutant regions. This suggests that a long-range extracellular molecule is not involved in inducing the formation of the EPL. On the other hand, wild-type columns had a disorganized IPL, suggesting that *Nr2e1* mutant cells can misdirect the neurites of wild-type cells. Wt↔*frc* chimeras also demonstrate that abnormal ganglion cell numbers per se are not the cause of IPL disorganization since they are restored in these chimeras.

The regulation of laminar targeting specificity in the retina depends on repulsive and attractive interactions mediated mostly by multiple short-range molecules [41–44]. A disrupted patterning of the IPL and disorganized amacrine branching is seen in *Pten*^*−/−*^ and *Dscam*^*−/−*^ mice [45, 46]. *Pten*^*−/−*^ mouse retinas have an expanded IPL with scattered cell bodies of abnormal morphology and clusters of dendrites in the IPL [45, 47]. Since *Pten* is a direct target of *Nr2e1*, its abnormal levels in *Nr2e1*^*frc/frc*^ retina could contribute to aberrant neurite organization. *Pten* remains expressed in the INL, IPL and GCL at P7 [45]. Whether *Nr2e1* is expressed in immature amacrine cells during development deserves further investigation.

Interestingly, a clear single ectopic layer in the INLhas been shown only in mice lacking the transmembrane repellents Sema5A and Sema5B [43] and the atypical cadherin Fat3 [41]. *Fat3*^*−/−*^ amacrine cells develop a second dendritic tree that projects away from the IPL and forms an additional synaptic layer in the INL [41]. Thus, our work suggests the interesting hypothesis that the Sema5 or Fat3 pathways may be affected in *Nr2e1*^*frc/frc*^ retinas.

The mechanisms by which signaling molecules orchestrate the development of a normal neuropil in the nervous system are still vastly unknown. Understanding how the lack of a transcription factor, such as *Nr2e1*, allows the formation of an ectopic neuropil in the retina would help to shed light into the cellular and molecular mechanisms leading to this process.

In summary, we found that Nr2e1 has numerous roles beyond preventing premature cell cycle exit in RPCs during retinal development including neurite organization and terminal cell differentiation. Our results show that Nr2e1 regulates the organization of inner retina neurites to the IPL and the development of neuronal subtypes, such as S-cones and glycineric amacrines as well as Müller glia. In addition, Nr2e1 regulates Müller glia differentiation cell-autonomously. Moreover, we have shown that prevention of retinal dystrophy is not regulated cell-autonomously by Nr2e1. A model summarizing the defects in cell numbers and lamination of the P7 *Nr2e1*^*frc/frc*^ and chimeric retina is depicted in Fig. 12.

## Materials and methods

### Mouse strains, husbandry, and breeding

*Nr2e1*^*frc*^ mice harbor a spontaneous deletion of the entire coding region of *Nr2e1* with no transcripts detected in the brain. These mice are highly aggressive and display hypoplastic cerebrum, olfactory lobes and retina [17]. Mice for study were generated by crossing *Nr2e1*^*frc/+*^ heterozygote females, which were congenic (>N30) on the C57BL/6 J (B6) (JAX stock # 000664) background, to *Nr2e1*^*frc/+*^ heterozygote males, which were congenic (>N30) on the 129S1/SvImJ (129) (JAX stock # 002448) background, thus generating *Nr2e1*^*+/+*^ control and *Nr2e1*^*frc/frc*^ mutant littermates on the hybrid B6129F1 background [48]. Males harboring the *NR2E1-lacZ* reporter BAC (bEMS86) were used to study *NR2E1* expression in the postnatal retina; strain B6.129P2(Cg)-*Hprt*^*tm73(Ple142-lacZ)Ems*^, abbreviated here as *NR2E1*-*lacZ* (MMRRC stock # 032962-JAX) [29]. Males harboring a random insertion of multiple-linked copies of the enhanced green fluorescent protein (EGFP) under an ubiquitous promoter were used for embryonic stem cell (ESC) generation; strain B6-Tg(CAG-EGFP)1Osb/J, abbreviated here as B6-EGFP (N > 10; JAX Stock # 003291) [49]. Mice containing the *lacZ* transgene at the *ROSA26* locus were used as a source of host blastocysts; strain 129S-*Gt(ROSA)26Sor*/J (JAX Stock # 002292) [50], backcrossed to B6 (N2-3) and abbreviated here as B6-*R26*^*lacZ*^. Eye studies were carried out on mice of either sex.

Mice were kept in a pathogen-free animal facility at the Centre for Molecular Medicine and Therapeutics, University of British Columbia (Vancouver, BC, Canada) on a 6 am to 8 pm light cycle with, 20 ± 2 °C, 50 % ± 5 % relative humidity, and food and water ad libitum. All procedures involving animals were in accordance with the Canadian Council on Animal Care (CCAC) and UBC Animal Care Committee (ACC) (Protocol numbers A11-0370 and A11-0081).

### Generation of embryonic stem cells (ESCs)

To obtain ESCs that contained both the *Nr2e1*^*frc*^ allele and the EGFP transgene as a marker, a two generation cross was performed. First, B6-*Nr2e1*^*frc/+*^ mice were crossed to B6-EGFP/0 mice to obtain B6-*Nr2e1*^*frc/+*^ EGFP/0 females. Then, these females were crossed to 129-*Nr2e1*^*frc/+*^ males to obtain B6129F1-*Nr2e1*^*frc/frc*^ EGFP/0 blastocysts. ESC lines were derived as previously described [51]. Briefly, blastocysts at 3.5 days post fertilization (dpf) were cultured in KSOM + AA media (Caisson Laboratories, North Logan, UT) under oil at 37 °C for 3–5 h. Then, each blastocyst was transferred to a single (1 x) 96-well containing mitomycin-C-inactivated mouse embryonic fibroblasts (MEFs) and cultured in KSR-ESC media (Knockout™ D-MEM with 2 mM l-glutamine, 0.1 mM MEM nonessential amino acid solution, 16 % Knockout™ Serum Replacement (all from Life Technologies Inc., Burlington, ON), and 1000 U/ml ESGRO® (LIF) (Millipore, Temecula, CA) . Once the blastocyst hatched and had a well-defined inner cell mass, it was trypsinized and transferred to 1 x 24-well containing inactivated MEFs. Cells were replated 1:1 if necessary. When cells reached confluence, they were split into 3 x 24-wells in 100 % ESC media. All 3 wells were combined at confluence and frozen in 3 separate vials until needed.

### Generation of chimeras

Chimeras were derived by microinjection of ESC into host blastocysts as previously described [51]. Either B6129F1-*Nr2e1*^*+/+*^ EGFP/0 or B6129F1-*Nr2e1*^*frc/frc*^ EGFP/0 ESCs were microinjected into B6-*R26*^*lacZ/+*^ host blastocysts. The generated chimeras were thus comprised of blastocyst-derived cells that were wild-type for *Nr2e1* and harbored the *R26*^*lacZ*^ transgene and ESC-derived cells that were either wild-type or mutant for *Nr2e1*, and harbored the EGFP transgene. A chimera is defined by the genotype of the blastocyst-derived and ESC-derived cells (blastocyst↔ESC): (*Nr2e1*^*+/+*^, *R26*^*lacZ/+*^) ↔ (*Nr2e1*^*+/+*^, EGFP/0) or (*Nr2e1*^*+/+*^, *R26*^*lacZ/+*^) ↔ (*Nr2e1*^*frc/frc*^, EGFP/0); and abbreviated as Wt↔*+/+* or Wt↔*frc/frc*, respectively. Two *Nr2e1*^*+/+*^ (mEMS4919 and mEMS4926) and two *Nr2e1*^*frc/frc*^ (mEMS4914 and mEMS4922) ESC lines were used to generate chimeras. After injection, blastocysts were implanted into the uterine horns of B6 2.5 day pseudopregnant females. Four Wt↔*+/+* and four Wt↔*frc/frc* P7 chimeric eyes were included in the study. Nine Wt↔*+/+* and ten Wt↔*frc/frc* P21 adult chimeric eyes were also included.

### Assessment of chimerism

Chimerism was initially assessed by coat color. Then, in slides prepared of the retina, chimerism was assessed by measuring the area of EGFP epifluorescence signal in the inner nuclear layer (INL) plus outer nuclear layer (ONL) using the software ImageJ [52]. The percentage of this fluorescence was assessed over the total INL plus ONL area.

### Histology

Eyes were fixed by intracardial perfusions performed on avertin-anesthetized mice with 4 % paraformaldehyde (PFA) in phosphate buffered saline (PBS). Eyes were then post-fixed in 4 % PFA for 30 min prior to cryoprotection in 25 % sucrose-PBS overnight. Subsequently, eyes were embedded in optimal cutting temperature (OCT) medium, cryosectioned at 12 μm and mounted on SuperFrost Plus slides (Thermo Fisher Scientific, Waltham, MA, USA). For β-galactosidase (β-gal) immunohistochemistry, staining was performed using X-gal substrate (5-bromo-4-chloro-3-indolyl-beta-D-galacto-pyranoside) for 18 h at 37 °C. To evaluate retinal thickness, retinal sections were subjected to hematoxylin and eosin staining. Briefly, tissue was incubated in hematoxylin for 5 min, washed in tap water and incubated in 1 % lithium carbonate solution for 30 sec. After washing in tap water again, the tissue was incubated in acid alcohol (1 %) for 5 s followed by another tap water wash and incubation in eosin Y solution for 5 min. After a final tap water wash, tissue was dehydrated in a gradient of ethanol, and xylene before mounting for microscopy. For immunofluorescence, at least three *Nr2e1*^*+/+*^ and three *Nr2e1*^*frc/frc*^ retinal sections per epitope were incubated in blocking solution (5 % bovine serum albumin, 0.3 % Triton X-100 in PBS) for 1 h. Subsequently, sections were incubated in primary antibody in blocking solution at 4 °C overnight. After three washes of 10 min in PBS, sections were incubated in secondary antibody in blocking solution with the DNA dye Hoechst-33342 for 1 h. Sections were washed 3 times in PBS and mounted in ProLong® Gold Antifade Reagent (Life Technologies Inc., Carlsbad, CA, USA). At least three transverse retinal sections were immunostained for each antibody using at least three eyes per genotype for imaging analysis.

### Antibodies

To label ganglion cells, we stained the retinas with antibodies to Brn3a, which is expressed in approximately 80 % of these cells [31]. For amacrines we used antibodies to the pan-amacrine markers Pax-6, and syntaxin-1A, which label amacrine cells and horizontal cells in the inner nuclear layer (INL) and amacrine and ganglion cells in the ganglion cell layer (GCL) [53, 54]. To visualize horizontal cells we used antibodies to calbindin, which is expressed in horizontal cells and a subpopulation of amacrine cells [55]. We used for: cones, cone-arrestin (arrestin-C) and S-Opsin antibodies that at P7 label the cell body, processes and outer segment of cones, which are located throughout the outer nuclear layer [56]; rods, rhodopsin antibody; bipolar cells, CHX10 antibody [53]; and Müller glia, Sox9 antibody [57]. The following primary antibodies were used: mouse anti-Brn3a 14A6 from Santa Cruz Biotechnology, Inc. (Dallas, TX, USA); mouse anti-Pax-6, anti-ISL1/2; rabbit anti-Pax-6 from Coavance (Princeton, NJ, USA); mouse anti-calbindin and anti-syntaxin from Sigma-Aldrich (St. Louis, MO, USA); mouse anti-Rhodopsin (ID4) from Dr. R.S. Molday, University of British Columbia (Vancouver, BC, Canada); sheep anti-Chx10 from Exalpha Biologicals, Inc. (Shirley, MA, USA); rabbit anti-cone-arrestin, rabbit anti-Sox9, rabbit anti-S-opsin, rabbit anti-mGluR1, goat anti-calretinin, chicken anti-vimentin, and guinea pig anti-GABA from Millipore (Billerica, MA, USA); mouse anti-PKCalpha, chicken anti-β-galactosidase, and rabbit anti-SOX-2 and anti-GlyT1 from Abcam (Cambridge, England); and mouse anti-GFAP from New England Biolabs (Ipswich, MA, USA).

### Imaging and cell counting

For dual visualization of X-gal blue precipitate and EGFP fluorescence in chimeras, images were taken with the Olympus BX61 motorized microscope using both DP Controller and In Vivo software (Olympus Corporation of the Americas, Center Valley, PA, USA). All remaining fluorescent images were taken with a Leica TCS SP5 II confocal microscope (Leica Microsystems, Wetzlar, Germany). To assess the numbers of each cell type in the retina, images were taken at 200 times magnification throughout a 12 μM retinal section and tiled. At least five equidistant transverse retinal sections throughout the entire eye were imaged: two images were close to the edge; two close to the middle half; and one central that included the optic nerve. One eye, from three different mice of each genotype, was included. Labeled cells were manually counted using the Cell Counter tool of ImageJ. Images were taken with a scan size of at least 1024 X 1024 pixels using 200 times magnification and visualized at 18 X 18 cms for accurate counting. The retinal marker channels and blue channel (Hoeschst stained-nuclei) images were superimposed followed by alternative switching between the channels to make sure individual cells were counted and to assess co-localization. Using the Cell Counter Tool a dot was placed on each cell counted to avoid double counting or miscounting of cells. Cell numbers were normalized to retinal length (μm). This length was assessed by drawing a line from edge to edge of retinal tissue along the nuclear layer where the cell-type to be counted was located. To count cells in chimeric retinas, marker-positive and EGFP-negative singled-labeled cells or marker-positive and EGFP-positive double-labeled cells were counted throughout a central retinal section. Cell numbers were normalized to retinal area. The EGFP-positive area was assessed by measuring the area of EGFP epifluorescence signal in the INL plus ONL using ImageJ. The EGFP-negative area was assessed by subtracting the EGFP positive area from the total INL plus ONL area. Cell density was expressed as the number of single-labeled cells over the EGFP-negative area or double-labeled cells over the EGFP-positive area. One eye, from three different chimeras for each ESC genotype, was included; totaling 6 eyes. Only chimeras with a contribution of >46 % mutant cells were counted. Quantification of retinal structural defects where INL and ONL cells overlap was performed in 3 eyes per genotype and 5 retinal sections as explained above for retina cell number quantification. A structural defect was counted when INL and ONL cells where in such proximity to each other that the OPL was no longer evident.

### Funduscopy

To assess the number of retinal blood vessels, funduscopy was performed as previously described [29, 58]. Briefly, eyes were dilated with 1 % atropine in PBS and photographed after 30 min. Animals were manually restrained without sedation.

### Image processing

Images were processed using ImageJ and Adobe Photoshop (Adobe Systems Incorporated**,** San Jose, CA, USA). Brightness, contrast, and scaling adjustments were performed as necessary.

### Statistical analyses

Statistical analyses were performed using Microsoft Excel (Microsoft Corporation, Redmond, Washington, USA) and XLSTAT (Addinsoft, New York, NY, USA). Student’s *t-*test was used to compare total cell numbers using raw data. In some cases, data was graphed as percentage of wild-type cells. Results were considered significance with a *P-*value ≤ 0.05. The standard error of the mean (SEM) was indicated with error bars. Z-scores were calculated as (retinal thickness or blood vessel number of Wt↔frc chimeras) minus ((mean of Wt↔Wt chimeras) divided by (standard deviation of Wt↔Wt chimeras)).

## Additional files

Additional file 1: Figure S1. Labeling of blastocyst-derived and ESC-derived cells was mutually exclusive in chimeras. (A) Embryonic stem cell (ESC) lines carrying wild-type and *Nr2e1*-mutant alleles plus the ubiquitously expressed EGFP transgene, were generated from E3.5 blastocysts derived from a two generation cross; first between B6-*Nr2e1* ^*frc/+*^ and B6-Tg(CAG-EGFP)1Osb/J/0 (abbreviated here as B6-EGFP/0) mice, and then B6-*Nr2e1* ^*frc/+*^ EGFP/0 and 129-*Nr2e1* ^*frc/+*^ mice. (B) ESCs were injected into B6-*Gt(ROSA)26Sor*/J/+ (abbreviated here as B6-*R26* ^*lacZ/+*^) E3.5 blastocysts containing the ubiquitously expressed *lacZ* gene, which encodes the enzyme β-galactosidase (β-gal). The resulting chimeras, denoted as blastocyst↔ESC [Wt↔Wt or Wt↔*frc*], contained wild-type (Wt) blastocyst-derived cells expressing β-gal and ESC-derived cells expressing EGFP. The latter cells were either wild-type or mutant (*frc*) for *Nr2e1*. (C) To demonstrate that β-gal and EGFP were cell-specific markers, retinal sections from B6-*R26* ^*lacZ/+*^ and B6-EGFP/0 control mice at P7 and P21 were incubated with X-gal. The distribution of X-gal product (blue) and EGFP epifluorescence signal (green) is shown. The EGFP signal was not affected by the X-gal reaction. (D) To demonstrate that β-gal and EGFP were cell-specific markers in chimeras, Wt↔Wt and Wt↔*frc* chimeric retinal sections from P7 and P21 mice were treated as in C. The labeling of blastocyst-derived (β-gal positive, blue) and ESC-derived (EGFP positive, green) cells was mutually exclusive. *n* = 4 for P7 Wt↔Wt, *n* = 9 for P21 Wt↔Wt, *n* = 4 for P7 Wt↔Wt, *n* = 10 for P21 Wt↔*frc*. Additional file 2: Figure S2.
*Nr2e1* ^*frc/frc*^ P7 retinas had reduced numbers of rod and bipolar cells that were not rescued in Wt↔*frc* chimeras. Transverse retinal sections from P7 *Nr2e1* ^*+/+*^, *Nr2e1* ^*frc/frc*^, and chimeric mice were immunostained for rhodopsin (rods) and CHX10 (bipolars). (A) rods (green) .(B) bipolar cells (red). (C) Each retinal cell type was counted throughout the retina as described in methods.. Reduced numbers of both rods and bipolar cells were observed in *Nr2e1*-mutant retinas compared to wild type (34 % and 27 % decrease, respectively). (D, E) The density of (D) rod and (E) bipolar cells (red) that were EGFP positive (green) appeared similar to the density of the corresponding EGFP negative cells in Wt↔Wt chimeras but looked lower in Wt↔frc chimeras. Representative images of a 46 % and a 71 % Wt↔Wt, and 62 % and 58 % Wt↔frc chimeric retina are shown for rods and bipolar cells, respectively. The arrows in E show a region comprised mostly of wild-type cells (EGFP negative) with a higher density of bipolar cells. (F, G) Quantification of the density of rod and bipolar cells that were derived from host blastocyst or ESCs in chimeras was assessed by counting single-labeled cells (marker positive and EGFP negative) or double-labeled cells (marker positive and EGFP positive) and dividing each by the EGFP negative or EGFP positive retinal area (ONL + INL), respectively. The density of *Nr2e1* ^*frc/frc*^ EGFP positive (F) rods and (G) bipolar cells was significantly lower (50 % and 53 % decrease, respectively) compared to the density of the corresponding EGFP negative cells in Wt↔*frc* chimeras. *n* = 3 for *Nr2e1* ^*+/+*^, *n* = 3 for *Nr2e1* ^*frc/frc*^, *n* = 3 for Wt↔Wt, *n* = 3 for Wt↔*frc*; *, P ≤ 0.05; ns, not significant; error bars represent SEM. GCL, ganglion cell layer; Hoechst, nuclear counterstain (blue); INL, inner nuclear layer; neg., negative; ONL, outer nuclear layer; pos., positive; scale bar = 50 μm. Additional file 3: Figure S3 Increased numbers of *Nr2e1* ^*frc/frc*^ GlyT1 positive cells were not rescued in P7 Wt↔*frc* chimeras. Transverse retinal sections from P7 chimeric mice were immunostained for the amacrine marker GlyT1. In Wt↔Wt chimeras, the density of GlyT1 amacrine cells that were EGFP positive (green) appeared similar to the density of EGFP negative amacrine cells. In Wt↔*frc* chimeras, the density of GlyT1 amacrine cells that were EGFP positive (green) appeared higher than the density of EGFP negative amacrine cells. Representative images of a 51 % Wt↔Wt and a 62 % Wt↔*frc* chimeric retina are shown. The arrow shows a region with high numbers of mutant cells (EGFP positive) where GlyT1 positive amacrine cells are overrepresented. GCL, ganglion cell layer; Hoechst, nuclear counterstain (blue); INL, inner nuclear layer; scale bar = 50 μm. Additional file 4: Figure S4. The ectopic inner plexiform layer of *Nr2e1* ^*frc/frc*^ retinas harbors neurites from various amacrine populations. Transverse retinal sections from P7 *Nr2e1* ^*+/+*^ and *Nr2e1* ^*frc/frc*^ mice were immunostained for calbindin and mGluR1. (A) Calbindin-positive amacrine cells (green) extended processes in the IPL of *Nr2e1* ^*+/+*^ and *Nr2e1* ^*frc/frc*^ retinas. In *Nr2e1* ^*frc/frc*^ retinas, some calbindin-positive cells also extended processes into the EPL (open arrow). (B) Magnification of the box in A showing those occasional ectopic calbindin-positive cell bodies (asterisks) that extended processes into the EPL (open arrow). (C) In wild-type retinas, mGluR1 (red) was expressed in some INL amacrine cell bodies immunostained for Pax-6 (green). In *Nr2e1*-mutant retinas, mGluR1 was also expressed in the ectopic EPL (open arrow). (D) Magnification of the box in E showing the expression of mGluR1 (solid arrow) in the *Nr2e1*-mutant EPL (open arrow) and occasional mGluR1 positive cell bodies that co-label with Pax-6 (asterisk) and extend processes into the EPL. The disorganized IPL of *Nr2e1* ^*frc/frc*^ retinas also harbored occasional amacrine cells bodies located between IPL sublamina (arrowhead). *n* = 3 for *Nr2e1* ^*+/+*^, *n* = 3 for *Nr2e1* ^*frc/frc*^; EPL, ectopic plexiform layer; GCL, ganglion cell layer; Hoechst, nuclear counterstain (blue); INL, inner nuclear layer; ONL, outer nuclear layer; OPL, outer plexiform layer; scale bar in A = 50 μm; scale bar in B = 22 μm. Additional file 5: Figure S5.
*Nr2e1* ^*frc/frc*^ amacrine cell numbers are normal in P21 Wt↔*frc* chimeras. Quantification of the density of syntaxin-1A positive cells that were derived from host blastocyst or ESCs in P21 chimeras was assessed by counting single-labeled cells (Syntaxin-1A positive and EGFP negative) or double-labeled cells (Syntaxin-1A positive and EGFP positive) and dividing them by the EGFP negative or EGFP positive retinal area (ONL + INL), respectively. In Wt↔ *frc* chimeras, the density of EGFP positive and EGFP negative syntaxin-1A cells was similar. *n* = 3 for Wt↔Wt, *n* = 3 for Wt↔*frc*; *, P ≤ 0.05; ns, not significant; error bars represent SEM; neg., negative; pos., positive. Additional file 6: Figure S6.
*Nr2e1* was expressed in Müller glia throughout postnatal development. Transverse retinal sections from P3, P7, P14, and P21 retinas from *Nr2e1* ^*+/+*^ mice containing a human *NR2E1*-driven *lacZ* reporter (*NR2E1-lacZ)* and *Nr2e1* ^*frc/frc*^ mice were immunostained for SOX-2 (red). This marker recognizes retinal precursor cells at earlier time-points, and an amacrine subpopulation and Müller glia at later time-points. (A) Sections from *NR2E1-lacZ* retinas were immunostained for β-galactosidase (β-gal). Co-localization of SOX-2 with β-gal in progenitors and Müller glia was observed at all time-points. Merge 1 shows SOX-2 (red) and β-gal (green) staining. Merge 2 shows Hoechst nuclear staining (blue) and SOX-2 staining (red). (B) In *Nr2e1* ^*frc/frc*^ retinas stained as in Merge 2, Müller glial density appeared comparable to wild type. *n* = 3 for *Nr2e1* ^*+/+*^, *n* = 3 for *Nr2e1* ^*frc/frc*^; GCL, ganglion cell layer; INL, inner nuclear layer; ONL, outer nuclear layer; scale bar = 50 μm. Additional file 7: Figure S7.
*Nr2e1* ^*frc/frc*^ Müller glia express Brn3a throughout the cell body. Magnification of a region of figure 11D (rectangle) showing a Müller cell displaying a typical irregular soma labeled with Brn3a and, as expected, not labeled with Vimentin. Distal processes towards the OPL, a stalk towards the GCL and projections that normally surround ganglion cell soma (end-feet) are labeled with both Brn3a and Vimentin. Müller glia lateral projections in the EPL are also evident with Brn3a but are not labeled with Vimentin. EPL = ectopic plexiform layer; GCL, ganglion cell layer; INL, inner nuclear layer; IPL = inner plexiform layer; ONL, outer nuclear layer; OPL = outer nuclear layer; scale bar = 50 μm.
