# Supplementary figures and images for: Nr2e1 regulates retinal lamination and the development of Müller glia, S-cones, and glycineric amacrine cells during retinogenesis
Source: Mol Brain. 2015 Jun 20;8:37. doi: 10.1186/s13041-015-0126-x (PMC4475312; doi:10.1186/s13041-015-0126-x)

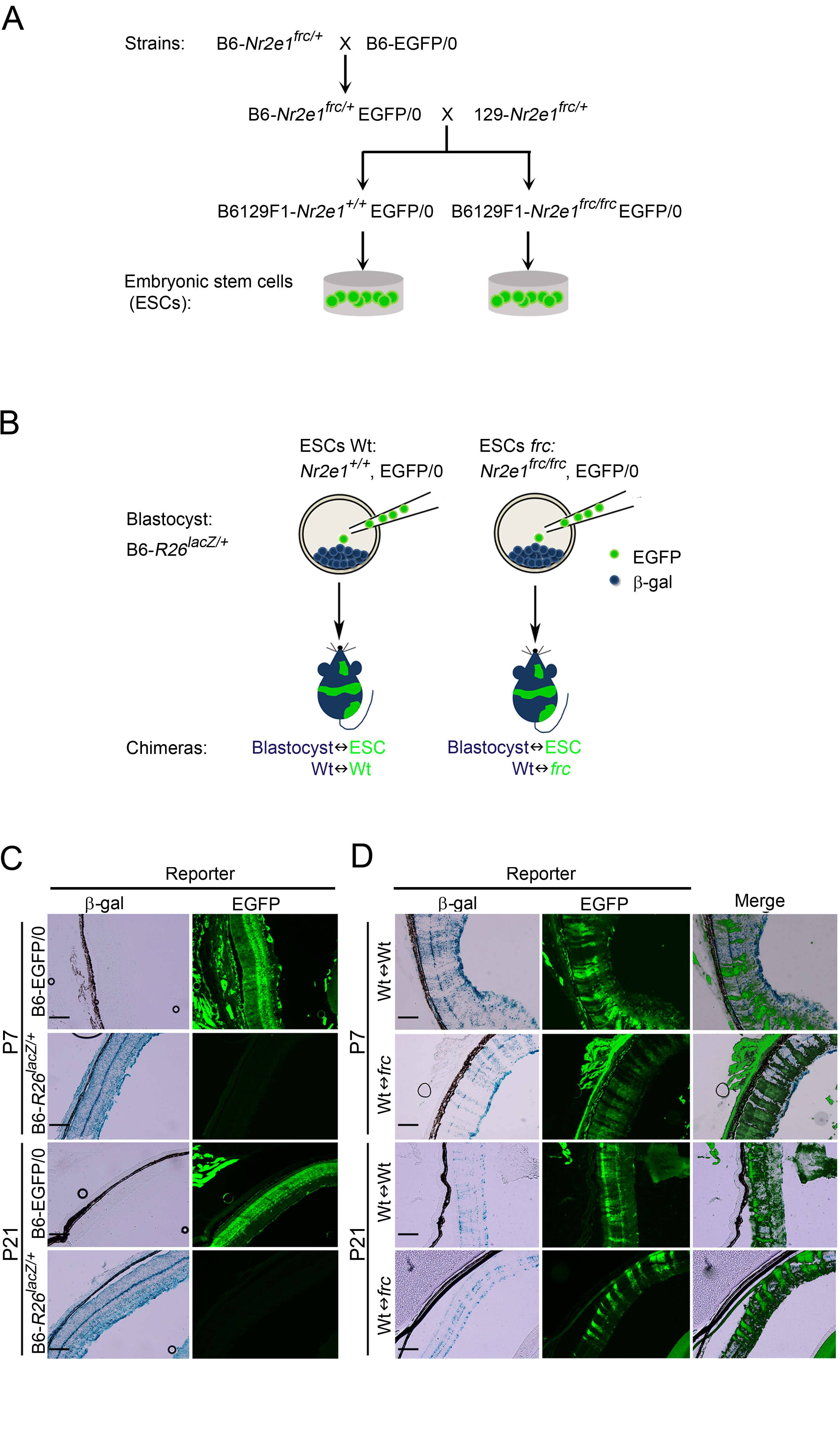

Supplement: Additional file 1: Figure S1. — Labeling of blastocyst-derived and ESC-derived cells was mutually exclusive in chimeras. (A) Embryonic stem cell (ESC) lines carrying wild-type and Nr2e1-mutant alleles plus the ubiquitously expressed EGFP transgene, were generated from E3.5 blastocysts derived from a two generation cross; first between B6-Nr2e1 frc/+ and B6-Tg(CAG-EGFP)1Osb/J/0 (abbreviated here as B6-EGFP/0) mice, and then B6-Nr2e1 frc/+ EGFP/0 and 129-Nr2e1 frc/+ mice. (B) ESCs were injected into B6-Gt(ROSA)26Sor/J/+ (abbreviated here as B6-R26 lacZ/+) E3.5 blastocysts containing the ubiquitously expressed lacZ gene, which encodes the enzyme β-galactosidase (β-gal). The resulting chimeras, denoted as blastocyst↔ESC [Wt↔Wt or Wt↔frc], contained wild-type (Wt) blastocyst-derived cells expressing β-gal and ESC-derived cells expressing EGFP. The latter cells were either wild-type or mutant (frc) for Nr2e1. (C) To demonstrate that β-gal and EGFP were cell-specific markers, retinal sections from B6-R26 lacZ/+ and B6-EGFP/0 control mice at P7 and P21 were incubated with X-gal. The distribution of X-gal product (blue) and EGFP epifluorescence signal (green) is shown. The EGFP signal was not affected by the X-gal reaction. (D) To demonstrate that β-gal and EGFP were cell-specific markers in chimeras, Wt↔Wt and Wt↔frc chimeric retinal sections from P7 and P21 mice were treated as in C. The labeling of blastocyst-derived (β-gal positive, blue) and ESC-derived (EGFP positive, green) cells was mutually exclusive. n = 4 for P7 Wt↔Wt, n = 9 for P21 Wt↔Wt, n = 4 for P7 Wt↔Wt, n = 10 for P21 Wt↔frc. [file 13041_2015_126_MOESM1_ESM.jpeg]

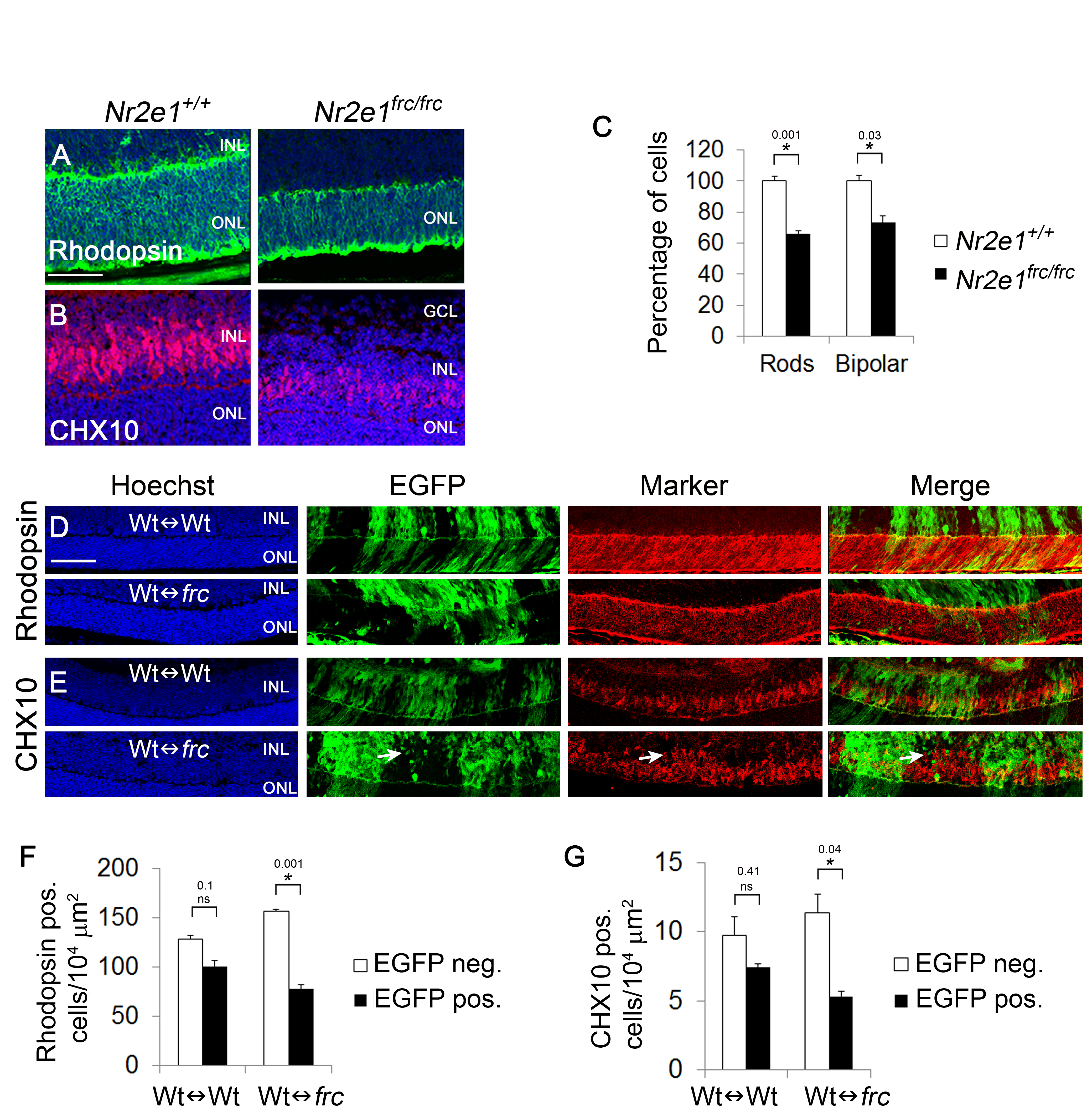

Supplement: Additional file 2: Figure S2. — Nr2e1 frc/frc P7 retinas had reduced numbers of rod and bipolar cells that were not rescued in Wt↔frc chimeras. Transverse retinal sections from P7 Nr2e1 +/+, Nr2e1 frc/frc, and chimeric mice were immunostained for rhodopsin (rods) and CHX10 (bipolars). (A) rods (green) .(B) bipolar cells (red). (C) Each retinal cell type was counted throughout the retina as described in methods.. Reduced numbers of both rods and bipolar cells were observed in Nr2e1-mutant retinas compared to wild type (34 % and 27 % decrease, respectively). (D, E) The density of (D) rod and (E) bipolar cells (red) that were EGFP positive (green) appeared similar to the density of the corresponding EGFP negative cells in Wt↔Wt chimeras but looked lower in Wt↔frc chimeras. Representative images of a 46 % and a 71 % Wt↔Wt, and 62 % and 58 % Wt↔frc chimeric retina are shown for rods and bipolar cells, respectively. The arrows in E show a region comprised mostly of wild-type cells (EGFP negative) with a higher density of bipolar cells. (F, G) Quantification of the density of rod and bipolar cells that were derived from host blastocyst or ESCs in chimeras was assessed by counting single-labeled cells (marker positive and EGFP negative) or double-labeled cells (marker positive and EGFP positive) and dividing each by the EGFP negative or EGFP positive retinal area (ONL + INL), respectively. The density of Nr2e1 frc/frc EGFP positive (F) rods and (G) bipolar cells was significantly lower (50 % and 53 % decrease, respectively) compared to the density of the corresponding EGFP negative cells in Wt↔frc chimeras. n = 3 for Nr2e1 +/+, n = 3 for Nr2e1 frc/frc, n = 3 for Wt↔Wt, n = 3 for Wt↔frc; *, P ≤ 0.05; ns, not significant; error bars represent SEM. GCL, ganglion cell layer; Hoechst, nuclear counterstain (blue); INL, inner nuclear layer; neg., negative; ONL, outer nuclear layer; pos., positive; scale bar = 50 μm. [file 13041_2015_126_MOESM2_ESM.jpeg]

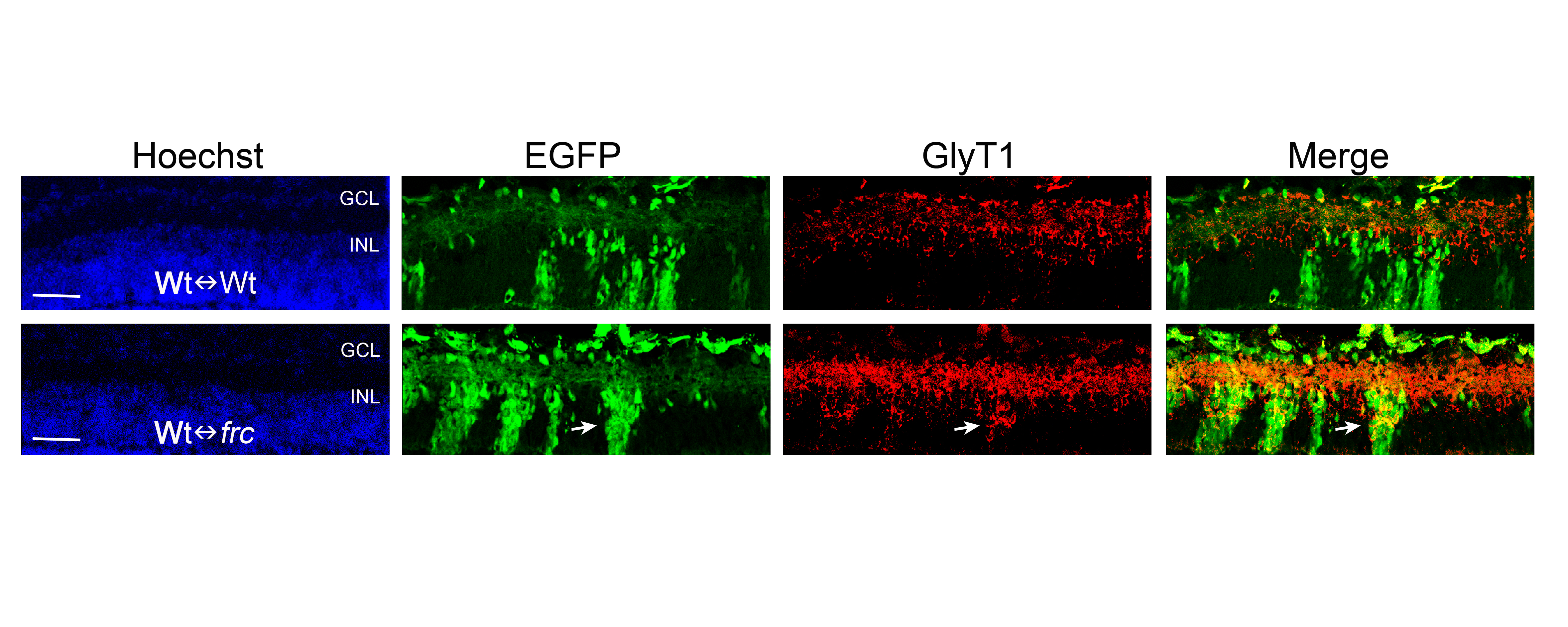

Supplement: Additional file 3: Figure S3 — Increased numbers of Nr2e1 frc/frc GlyT1 positive cells were not rescued in P7 Wt↔frc chimeras. Transverse retinal sections from P7 chimeric mice were immunostained for the amacrine marker GlyT1. In Wt↔Wt chimeras, the density of GlyT1 amacrine cells that were EGFP positive (green) appeared similar to the density of EGFP negative amacrine cells. In Wt↔frc chimeras, the density of GlyT1 amacrine cells that were EGFP positive (green) appeared higher than the density of EGFP negative amacrine cells. Representative images of a 51 % Wt↔Wt and a 62 % Wt↔frc chimeric retina are shown. The arrow shows a region with high numbers of mutant cells (EGFP positive) where GlyT1 positive amacrine cells are overrepresented. GCL, ganglion cell layer; Hoechst, nuclear counterstain (blue); INL, inner nuclear layer; scale bar = 50 μm. [file 13041_2015_126_MOESM3_ESM.jpeg]

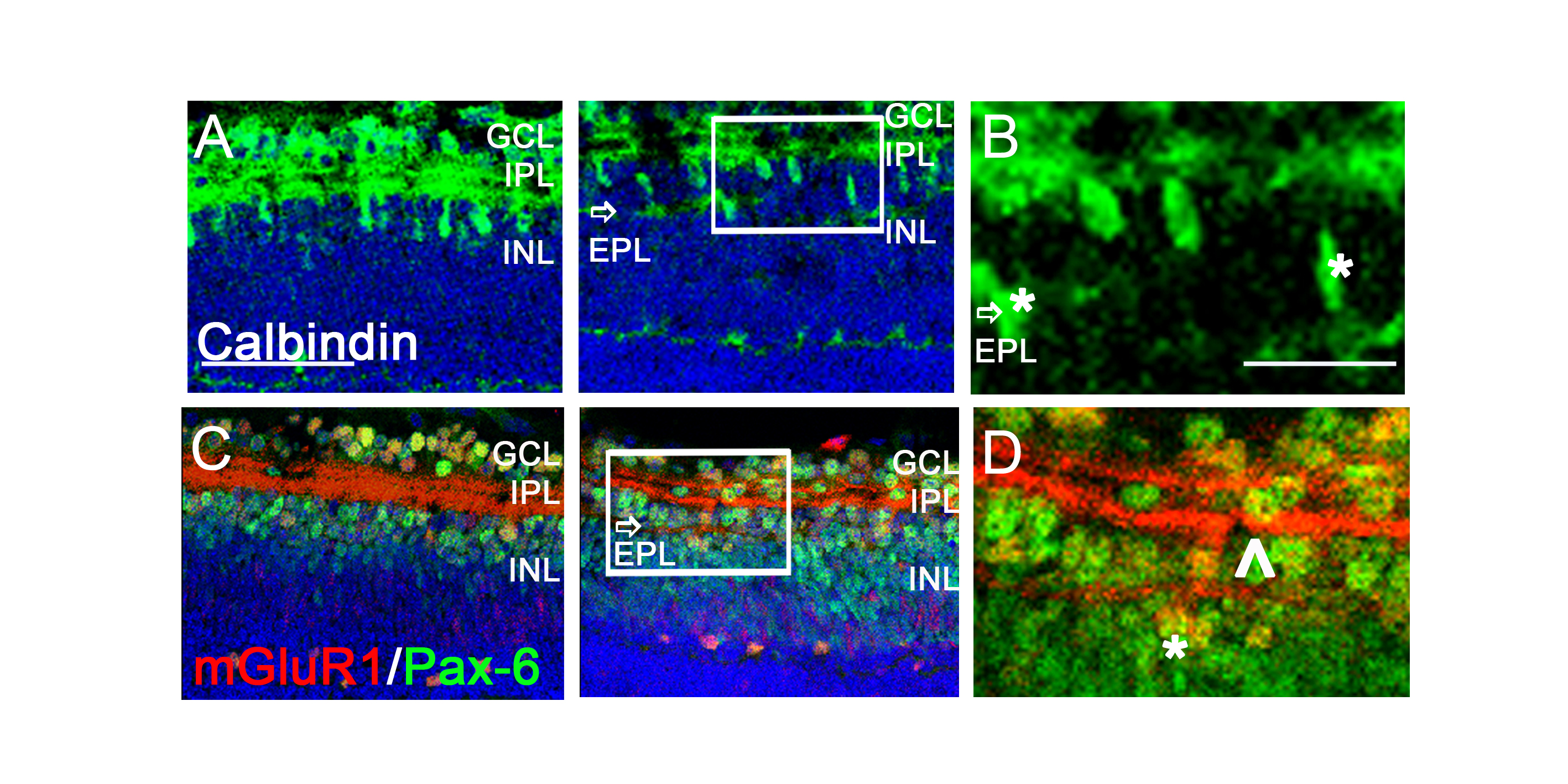

Supplement: Additional file 4: Figure S4. — The ectopic inner plexiform layer of Nr2e1 frc/frc retinas harbors neurites from various amacrine populations. Transverse retinal sections from P7 Nr2e1 +/+ and Nr2e1 frc/frc mice were immunostained for calbindin and mGluR1. (A) Calbindin-positive amacrine cells (green) extended processes in the IPL of Nr2e1 +/+ and Nr2e1 frc/frc retinas. In Nr2e1 frc/frc retinas, some calbindin-positive cells also extended processes into the EPL (open arrow). (B) Magnification of the box in A showing those occasional ectopic calbindin-positive cell bodies (asterisks) that extended processes into the EPL (open arrow). (C) In wild-type retinas, mGluR1 (red) was expressed in some INL amacrine cell bodies immunostained for Pax-6 (green). In Nr2e1-mutant retinas, mGluR1 was also expressed in the ectopic EPL (open arrow). (D) Magnification of the box in E showing the expression of mGluR1 (solid arrow) in the Nr2e1-mutant EPL (open arrow) and occasional mGluR1 positive cell bodies that co-label with Pax-6 (asterisk) and extend processes into the EPL. The disorganized IPL of Nr2e1 frc/frc retinas also harbored occasional amacrine cells bodies located between IPL sublamina (arrowhead). n = 3 for Nr2e1 +/+, n = 3 for Nr2e1 frc/frc; EPL, ectopic plexiform layer; GCL, ganglion cell layer; Hoechst, nuclear counterstain (blue); INL, inner nuclear layer; ONL, outer nuclear layer; OPL, outer plexiform layer; scale bar in A = 50 μm; scale bar in B = 22 μm. [file 13041_2015_126_MOESM4_ESM.jpeg]

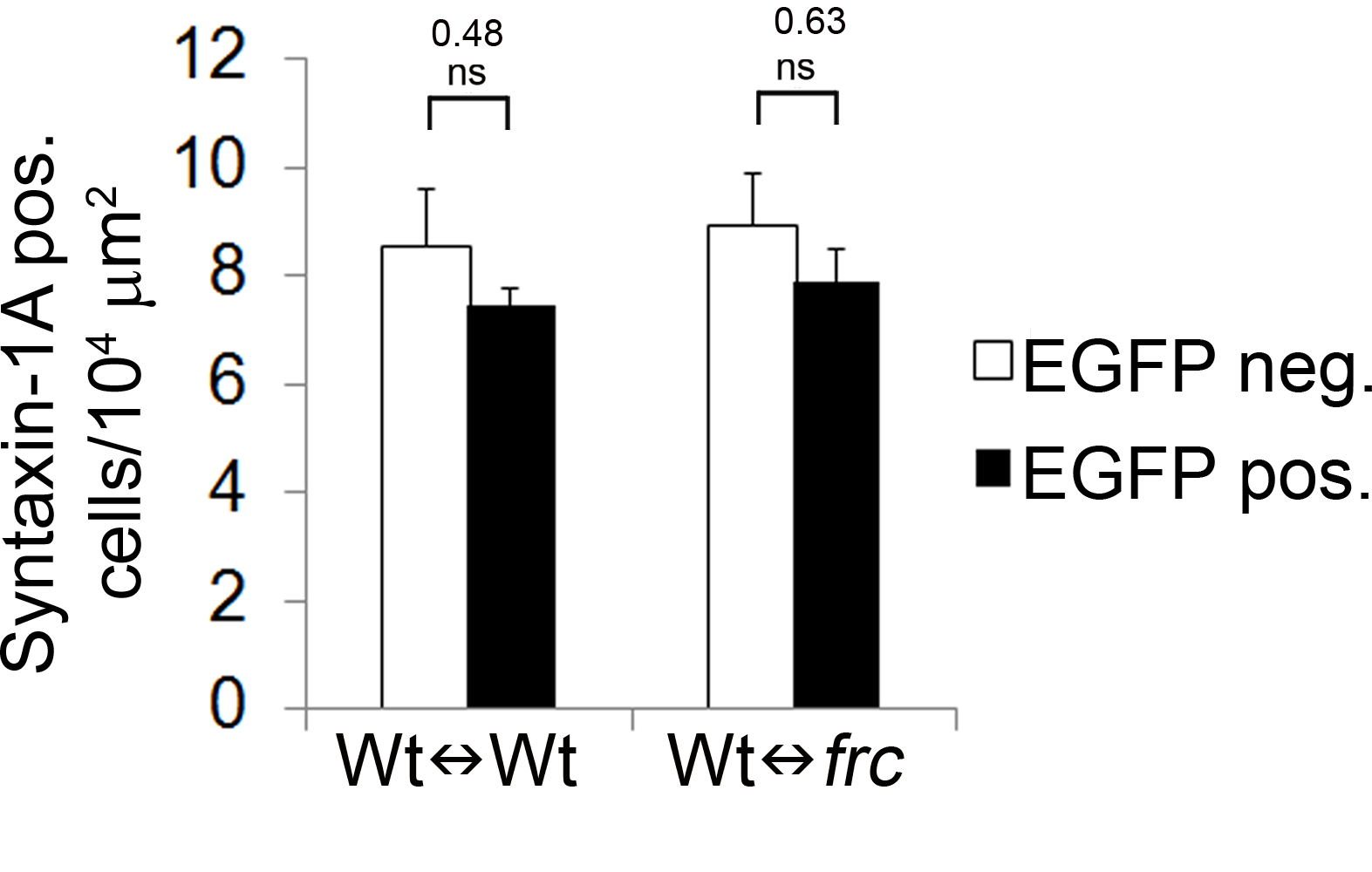

Supplement: Additional file 5: Figure S5. — Nr2e1 frc/frc amacrine cell numbers are normal in P21 Wt↔frc chimeras. Quantification of the density of syntaxin-1A positive cells that were derived from host blastocyst or ESCs in P21 chimeras was assessed by counting single-labeled cells (Syntaxin-1A positive and EGFP negative) or double-labeled cells (Syntaxin-1A positive and EGFP positive) and dividing them by the EGFP negative or EGFP positive retinal area (ONL + INL), respectively. In Wt↔ frc chimeras, the density of EGFP positive and EGFP negative syntaxin-1A cells was similar. n = 3 for Wt↔Wt, n = 3 for Wt↔frc; *, P ≤ 0.05; ns, not significant; error bars represent SEM; neg., negative; pos., positive. [file 13041_2015_126_MOESM5_ESM.jpeg]

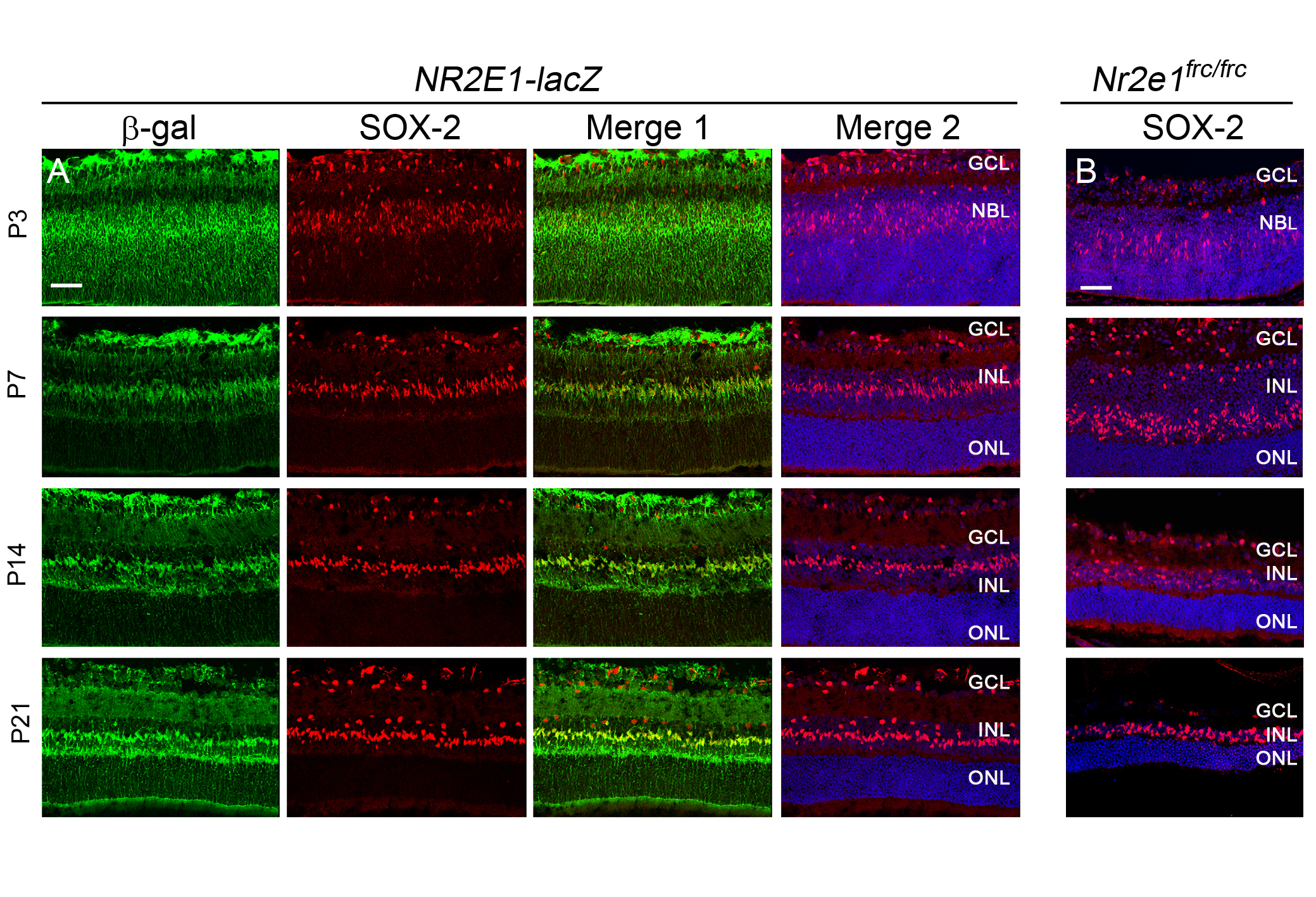

Supplement: Additional file 6: Figure S6. — Nr2e1 was expressed in Müller glia throughout postnatal development. Transverse retinal sections from P3, P7, P14, and P21 retinas from Nr2e1 +/+ mice containing a human NR2E1-driven lacZ reporter (NR2E1-lacZ) and Nr2e1 frc/frc mice were immunostained for SOX-2 (red). This marker recognizes retinal precursor cells at earlier time-points, and an amacrine subpopulation and Müller glia at later time-points. (A) Sections from NR2E1-lacZ retinas were immunostained for β-galactosidase (β-gal). Co-localization of SOX-2 with β-gal in progenitors and Müller glia was observed at all time-points. Merge 1 shows SOX-2 (red) and β-gal (green) staining. Merge 2 shows Hoechst nuclear staining (blue) and SOX-2 staining (red). (B) In Nr2e1 frc/frc retinas stained as in Merge 2, Müller glial density appeared comparable to wild type. n = 3 for Nr2e1 +/+, n = 3 for Nr2e1 frc/frc; GCL, ganglion cell layer; INL, inner nuclear layer; ONL, outer nuclear layer; scale bar = 50 μm. [file 13041_2015_126_MOESM6_ESM.jpeg]

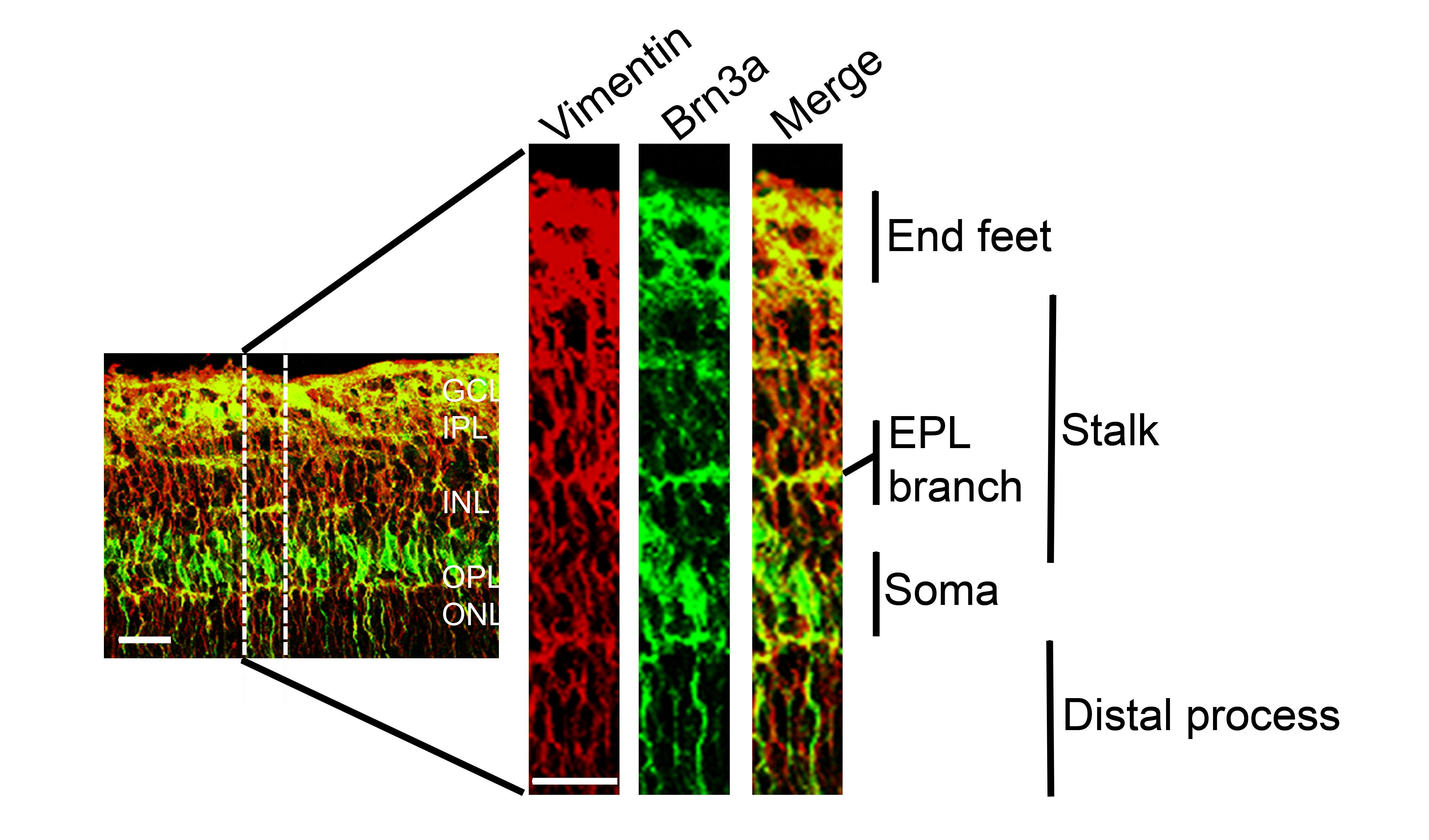

Supplement: Additional file 7: Figure S7. — Nr2e1 frc/frc Müller glia express Brn3a throughout the cell body. Magnification of a region of figure 11D (rectangle) showing a Müller cell displaying a typical irregular soma labeled with Brn3a and, as expected, not labeled with Vimentin. Distal processes towards the OPL, a stalk towards the GCL and projections that normally surround ganglion cell soma (end-feet) are labeled with both Brn3a and Vimentin. Müller glia lateral projections in the EPL are also evident with Brn3a but are not labeled with Vimentin. EPL = ectopic plexiform layer; GCL, ganglion cell layer; INL, inner nuclear layer; IPL = inner plexiform layer; ONL, outer nuclear layer; OPL = outer nuclear layer; scale bar = 50 μm. [file 13041_2015_126_MOESM7_ESM.jpeg]
